# Changes to balance dynamics following a high-intensity run are associated with future injury occurrence in recreational runners

**DOI:** 10.3389/fnetp.2023.1227861

**Published:** 2023-11-21

**Authors:** Mariana R. C. Aquino, Joshua J. Liddy, C. Dane Napoli, Sérgio T. Fonseca, Richard E. A. van Emmerik, Michael A. Busa

**Affiliations:** ^1^ Graduate Program of Rehabilitation Sciences, Department of Physical Therapy, Universidade Federal de Minas Gerais, Belo Horizonte, Brazil; ^2^ Department of Kinesiology, University of Massachusetts Amherst, Amherst, MA, United States; ^3^ Center for Human Health and Performance, Institute for Applied Life Sciences, University of Massachusetts Amherst, Amherst, MA, United States

**Keywords:** running injuries, fatigue, single-leg squat, physiological time series analysis, regularity, sample entropy, crossover phenomena, detrended fluctuation analysis

## Abstract

**Background:** Fatigue is associated with increased injury risk along with changes in balance control and task performance. Musculoskeletal injury rates in runners are high and often result from an inability to adapt to the demands of exercise and a breakdown in the interaction among different biological systems. This study aimed to investigate whether changes in balance dynamics during a single-leg squat task following a high-intensity run could distinguish groups of recreational runners who did and did not sustain a running-related injury within 6 months.

**Methods:** Thirty-one healthy recreational runners completed 60 s of single-leg squat before and after a high-intensity run. Six months after the assessment, this cohort was separated into two groups of 13 matched individuals with one group reporting injury within this period and the other not. Task performance was assessed by the number of repetitions, cycle time, amplitude, and speed. To evaluate balance dynamics, the regularity and temporal correlation structure of the center of mass (CoM) displacements in the transverse plane was analyzed. The interaction between groups (injury, non-injured) and time (pre, post) was assessed through a two-way ANOVA. Additionally, a one-way ANOVA investigated the percent change difference of each group across time.

**Results:** The injured group presented more regular (reduced entropy; 15.6%) and diffusive (increased short-term persistence correlation; 5.6%) CoM displacements after a high-intensity run. No changes were observed in the non-injured group. The within-subject percent change was more sensitive in demonstrating the effects of fatigue and distinguishing the groups, compared to group absolute values. No differences were observed in task performance.

**Discussion:** Runners who were injured in the future demonstrate changes in balance dynamics compared to runners who remain injury-free after fatigue. The single-leg squat test adopted appears to be a potential screening protocol that provides valuable information about balance dynamics for identifying a diminished ability to respond to training and exercise.

## 1 Introduction

Musculoskeletal injury is a multifactorial process (Bittencourt et al., 2016) commonly observed in recreational sports-participants (National Safety Council, 2022). Recreational runners exhibit high rates of injuries (19%–92%) (Lun et al., 2004; Hreljac, 2005; Van Gent et al., 2007; Buist et al., 2010; Videbæk et al., 2015; Messier et al., 2018; Mulvad et al., 2018) and common risk factors for running-related injuries are not reliable predictors of future injury (Ceyssens et al., 2019). Exercise-induced fatigue can elicit changes in balance and task performance, which are associated with injury risk (Corbeil et al., 2003; Gribble et al., 2004; Lin et al., 2009; Clansey et al., 2012; Zech et al., 2012; Schütte et al., 2018; Huygaerts et al., 2020; Verschueren et al., 2020; Heil and Büsch, 2022). There have been proposals that sports injuries result from changes to interactions among musculoskeletal structures and neurophysiological systems that indicate the inability to withstand the demands of exercise (Fonseca et al., 2020). Therefore, rather than identifying individual risk factors, it may be more valuable to examine macroscopic variables that reflect interactions between many self-organizing elements to gain insights into future injury status (Balagué et al., 2020; Fonseca et al., 2020). Fatigue-inducing exercise, such as a high-intensity run, may elicit changes in the dynamics of these macroscopic variables which precede reductions in task performance or injury (Pol et al., 2018). Therefore, there is a need to identify and examine changes in variables that indicate the ability to resist exercise-induced fatigue and the potential incidence of future running injury (Balagué et al., 2020).

Balance control, which refers to the maintenance of upright posture in relation to external forces and the corresponding changes in posture that facilitate task performance, is negatively impacted by fatigue-inducing exercise (Corbeil et al., 2003; Vuillerme and Hintzy, 2007; Springer and Pincivero, 2009; Zech et al., 2012; Monjo et al., 2015; Verschueren et al., 2020; Heil and Büsch, 2022). Generally, measures of balance control examine center of pressure (CoP) or center of mass (CoM) movements, including time-independent (e.g., sway area) and time-dependent measures (e.g., entropy), the latter of which provide information about balance dynamics. Balance dynamics consist of transitions between postures necessary to complete the task and patterns of variability that emerge from perception-action coupling (e.g., Riccio and Stoffregen, 1988; Riccio, 1993; Van Wegen et al., 2002; Carpenter et al., 2010; Murnaghan et al., 2014). Because these emergent movement dynamics may provide insights into the adaptative capacity of sports participants (Van Emmerik et al., 2016; Fonseca et al., 2020), this study investigated balance dynamics in recreational runners.

Single-leg tests, including the single-leg squat and single-leg step-down, are used to assess neuromuscular control in sports-participants (Bittencourt et al., 2012; Paterno et al., 2015; Ugalde et al., 2015; Cardoso et al., 2021). The single-leg squat task requires coordination across multiple joints to maintain balance with a reduced base of support while raising and lowering the body (Bittencourt et al., 2012; Ksoll et al., 2022), and has been used to screen for injury risk and readiness to return to play, and distinguish currently injured from non-injured individuals (Ugalde et al., 2015; Cardoso et al., 2021; Petushek et al., 2021). However, single-leg squat performance is often evaluated from a limited number of repetitions (Bittencourt et al., 2012; Rees et al., 2019; Cardoso et al., 2021; Ksoll et al., 2022), which do not provide enough information to distinguish sports participants who eventually become injured from those who do not.

The CoM is a useful variable for understanding the performance and balance components of the single-leg squat task. Performance can be assessed by the number of repetitions and spatiotemporal movement parameters (e.g., cycle time and amplitude), which are commonly used to examine the effects of fatigue, injury risk, and return to play (Burnham et al., 2016; Cardoso et al., 2021; Heil and Büsch, 2022). Because the task requires repeated raising and lowering of the body, the vertical displacement of the CoM can provide insights into task performance. By contrast, balance dynamics can be assessed by examining the time-varying patterns of movements in the transverse plane, which have implications for ensuring mechanical stability and simultaneously facilitating task performance. Therefore, this study investigated the CoM displacements in the vertical direction (performance) and transverse plane (balance) during a prolonged single-leg squat task.

Current approaches for predicting injuries in sports participants are limited for several reasons. One problem is that assessing performance at a single point in time may mask insights into adaptive capacity (Fonseca et al., 2020). For instance, retrospective cross-sectional studies may produce equivocal findings by limiting their focus to group differences without understanding the directionality of changes over time. To prevent and mitigate future injuries, tools and concepts from dynamical systems theory, information theory, and complexity science may provide a better understanding of the dynamics of macroscopic behaviors that emerge from a complex network of subsystems in response to perturbations, such as fatigue (Balagué et al., 2020; Fonseca et al., 2020). These system-level behaviors can be tracked and quantified to provide insights into the individual adaptative capacities in response to exercise and fatigue (Pol et al., 2018; Balagué et al., 2020). Another problem is that traditional physiological (e.g., VO_2_max) and performance measures (e.g., number of cycles) may be conserved even though interactions and synergies between many elements and physiological systems may change in advance of injury or disease (Balagué et al., 2020; Garcia-Retortillo and Ivanov, 2022). People exploit the many available solutions to complete tasks (Latash, 2012) and the organization of network interactions does not depend on the specific movement, but rather is associated with distinct physiological states (i.e., rest, exercise, fatigue) (Garcia-Retortillo and Ivanov, 2022). Therefore, people can accomplish the same task performance (e.g., number of cycles, cycle amplitude and time) with different movements (e.g., balance dynamics). Evaluating changes in task performance *and* balance dynamics may provide a more complete and potentially more sensitive means of understanding how runners cope with fatigue and may help identify individuals at risk of developing future injury.

Postural displacements of the CoM are characterized by stochastic fluctuations spanning a wide range of spatiotemporal scales (Collins and De Luca, 1993; Collins and De Luca, 1994; Duarte and Zatsiorsky, 2000; Delignières et al., 2011). Past studies have investigated fatigue-induced changes to balance (Lin et al., 2009; McGregor et al., 2011; Schütte et al., 2015; Vázquez et al., 2016) and differentiated injured and non-injured sports participants (Kiefer et al., 2013; Terada et al., 2015; Quirino et al., 2021) by examining regularity statistics and temporal correlation properties. Notably, the combined use of these measures can provide complementary insights into physiological signals with distinct time-varying properties (Liddy and Busa, 2023). For example, different temporal correlations (e.g., 0 < *α* < 1) can return similar entropy values, which can lead to misinterpretations of the signal dynamics (Liddy and Busa, 2023).

Entropy methods, such as Sample Entropy (SampEn), provide a continuum measure of the amount of randomness or uncertainty contained in a sequence of data (Richman and Moorman, 2000). Lower entropy, which indicates greater regularity, has generally been observed in populations with reduced adaptive capacity (Busa and Van Emmerik, 2016). For example, more regular balance dynamics have been associated with older adults, people with neurological disorders and injury history, when compared to young or healthy matched controls (Roerdink et al., 2006; Donker et al., 2008; Kiefer et al., 2013; Quatman-Yates et al., 2015; Busa and Van Emmerik, 2016). Furthermore, runners with a history of injury show more regular movement patterns when exposed to fatigue compared to runners without past injuries (Quirino et al., 2021). However, fatigue can also lead to less regular balance dynamics in healthy populations (McGregor et al., 2011; Schütte et al., 2015). Sports participants with greater future injury risk may show early signs of reduced capacity when exposed to fatigue, which elicits many of the same physiological and behavioral changes that emerge prior to injury (Pol et al., 2018). Thus, balance dynamics may not differ at baseline between runners who do and do not become injured. However, otherwise healthy runners who eventually become injured may demonstrate more regular balance dynamics following fatigue-inducing exercise.

Measures of temporal correlations, such as Detrended Fluctuation Analysis (DFA), provide insights into the (in)dependence of postural fluctuations by measuring variance over increasing windows of time. Postural displacements during quiet standing are usually characterized by short-term persistent (positive) correlations and long-term anti-persistent (negative) correlations (Collins and De Luca, 1993; Collins and De Luca, 1995a; Collins and De Luca, 1995b; Riley et al., 1997a; Riley et al., 1997b; Delignières et al., 2011), indicating local and global stationarity with distinct time-varying structures. The transition from persistent to anti-persistent correlations observed during quiet standing is referred to as a crossover, which more broadly refers to a qualitative change in the temporal correlation structure.

Short- and long-term correlation structures, as well as the crossover point, can change when different postural strategies are adopted to maintain balance or support suprapostural performance (Riley et al., 1997a; Riley et al., 1997b). For clarity, short-term and long-term temporal correlations can be understood relative to the crossover point rather than with respect to the neurophysiological processes involved in balance control. In the single-leg squat task, the long-term bounding of the CoM displacements will produce reversals that are more reflective of the task dynamics across cycles, whereas the short-term drifts reflect periods dominated by movement within cycles. Because task performance is not expected to differ between groups of runners who did and did not become injured, the expectation is that fatigue-related changes to balance dynamics will be restricted to the short-term correlation structure as opposed to the crossover point or long-term correlation structure.

The purpose of this study was to investigate whether changes to balance dynamics during a single-leg squat task following a high-intensity run differentiate recreational runners who did and did not sustain a running-related injury in the next 6 months. We also assessed task performance before and after the fatigue protocol. We made the following predictions: 1) task performance would not differ between injured and non-injured runners before or after the high-intensity run; 2) balance dynamics would also not differ between these groups before the high-intensity run; 3) fatigue-induced changes to balance dynamics in both groups, with injured runners expected to be more affected than non-injured runners; 4) specifically, entropy and short-term correlations, which are more representative of within-cycle dynamics related to balance control, would become more regular and persistent following fatigue 5) long-term correlations and the crossover point, which are more representative of between-cycle dynamics related to task performance, would not differ between groups.

## 2 Materials and methods

### 2.1 Participants

Thirty-two recreational runners were recruited for a longitudinal study, which included performing a modified single-leg squat task before and after a high-intensity running protocol with a six-month follow-up. Eligible participants were adult runners between 18 and 60 years old who reported completing a minimum of 20 km/week at least twice a week regularly for the past 6 months and were currently without injuries (Hreljac, 2005; Encarnación-Martínez et al., 2020). An injury was defined as musculoskeletal pain or discomfort resulting in training volume reduction or restriction for 7 days or three consecutive training sessions, or the need for healthcare professional assistance (Yamato et al., 2015). Participants were excluded if they could not complete the single-leg squat task or the running protocol or reported pain during evaluation. Only one participant was excluded after presenting pain during testing. The remaining participants completed a retrospective online survey 6 months after the experiment and reported whether they experienced an injury in the preceding 6 months. This study was approved by the Universidade Federal de Minas Gerais Ethics Committee, and all participants provided informed consent.

### 2.2 Experimental procedures

**FIGURE 1:**
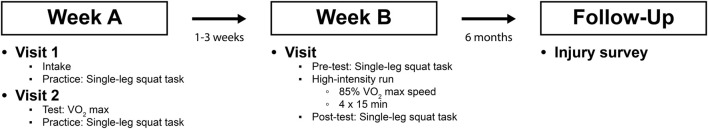
Experimental timeline. Two visits were completed during the first week of testing (Week A). During the first visit, intake information was collected, and participants practiced the single-leg squat task. During the second visit, participants completed VO_2_ max testing and additional practice of the single-leg squat task. The second week of testing (Week B) consisted of a single visit where participants completed pre- and post-tests of the single-leg squat task separated by a high-intensity running protocol. Six months later, participants completed a follow-up survey to retrospectively report injuries incurred since completing testing.

Participants were tested over separate weeks within 1 month (Figure 1). During the first week (Week A), participants completed two visits to practice the single-leg squat task and estimate their maximum volume of oxygen consumed (VO_2_ max), which was used to characterize aerobic fitness and normalize the intensity of the running protocol. Pilot data indicated that 2 days of practice were needed to minimize learning effects. Participants also reported their training routine, including average pace (min/km), average volume (km/week), running experience (years), additional sports or physical activities (e.g., strength training), and previous injuries. During the second week (Week B), participants performed the single-leg squat task before and after a high-intensity running protocol.

Six months later, participants completed a follow-up injury survey, where they reported whether they sustained an injury or not, as well as their average pace and weekly training volume over the past 6 months. Participants were stratified into two cohorts based on injury status. If they reported an injury, additional questions were used to probe when it occurred, the affected segment, structure, or muscle group. They also reported if they had to reduce or stop training and if they required healthcare professional assistance.

#### 2.2.1 Single-leg squat task

**FIGURE 2:**
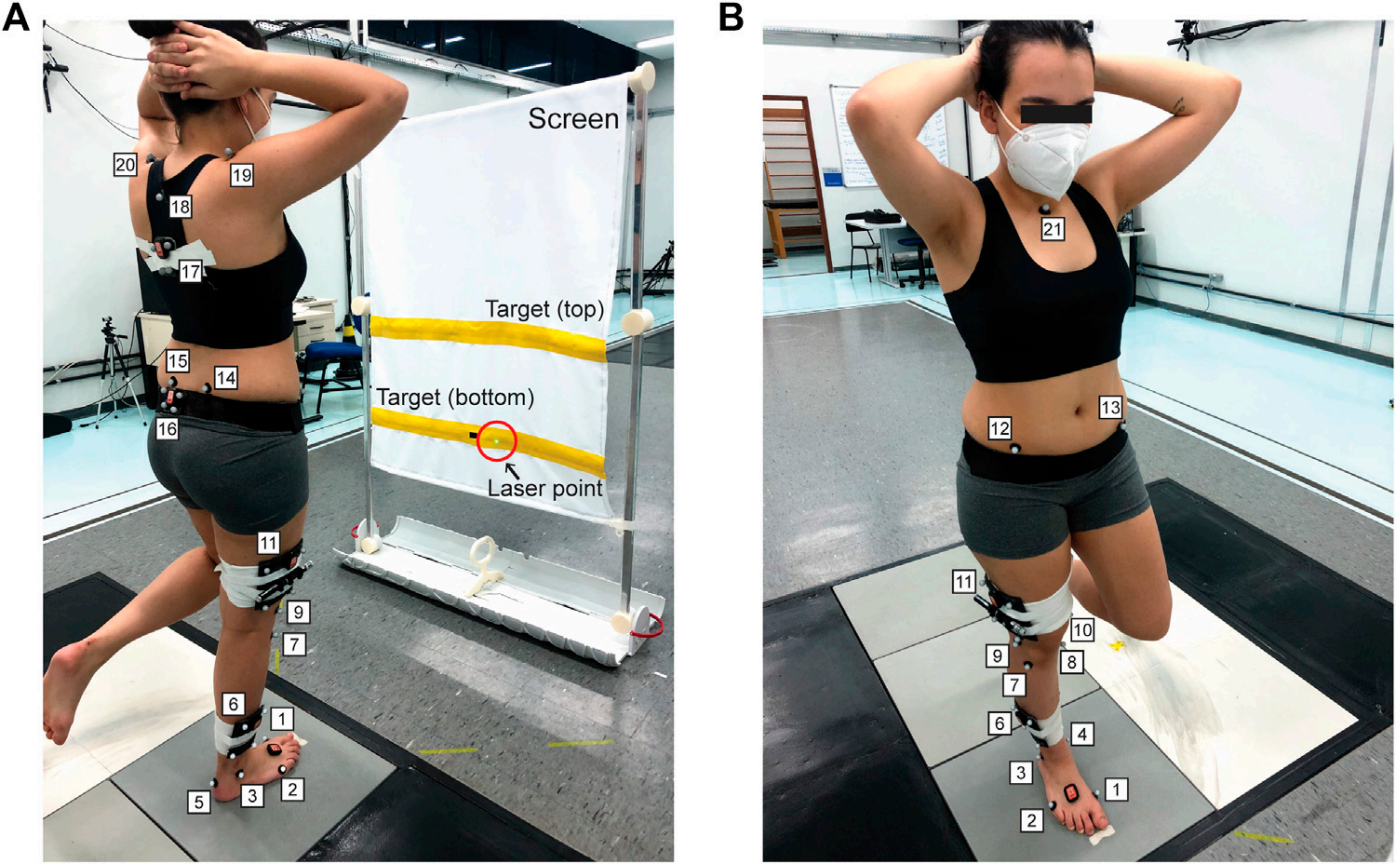
Single-leg squat task. **(A)** Rear view of a person performing the single-leg squat task. Participants stood barefoot on their dominant leg in front of a white, vertical screen with yellow horizontal targets. A laser was attached above the knee and projected onto the screen. Participants continuously moved the laser point back and forth between the bottom and top targets by completing squats. **(B)** Front view of the same participant. Markers were placed on the first metatarsal head (1), fifth metatarsal head (2), lateral and medial malleoli (3–4), calcaneus (5), shank (6), lateral and medial tibial condyles (7–8), lateral and medial femoral epicondyles (9–10), thigh (11), right and left anterior superior iliac spines (12–13), right and left posterior superior iliac spines (14–15), sacrum (16), trunk (17), posterior projection of jugular notch (18), right and left acromia (19–20), and jugular notch (21). Each cluster had four markers leading to 33 total markers.

The single-leg squat task was adapted from Cardoso et al. (2021). Participants stood barefoot on their dominant leg—defined as the self-reported leg preferred for kicking a ball—with their foot aligned perpendicular to a vertical screen located in front of them with their hands crossed behind their head (Figure 2; Supplementary Video S1). The screen contained two horizontally oriented targets separated vertically by 30 cm. A laser pointer was attached above the knee and oriented orthogonally to the longitudinal axis of the thigh. The vertical position of the bottom target was adjusted so that the laser was centered when the knee was in extension. The horizontal distance between the participant and the screen was adjusted to ensure that at least 40 degrees of knee flexion were required to complete each repetition. This knee flexion range was selected based on previous studies (Bittencourt et al., 2012; Ugalde et al., 2015; Rees et al., 2019; Petushek et al., 2021) and our pilot study, so that it was challenging but feasible to perform an uninterrupted bout of single-leg squats for 60 s.

To complete the task, participants continuously performed single-leg squats for 60 s by flexing and extending at the ankle, knee, and hip while keeping the trunk upright and the contralateral foot off the ground. Participants were instructed to repeatedly move the laser between the bottom and top targets at their preferred speed. They were further instructed not to reverse direction until the laser had contacted or exceeded the target. Rate of perceived exertion (RPE) was recorded before and after each test using a modified Borg RPE scale with values of 0–10 (Borg, 1998; Impellizzeri et al., 2004; Bellenger et al., 2019). The task was stopped and repeated if the participant moved their hands or dominant foot out of position, or if they touched the ground or support leg with their non-dominant leg. If the pre-test needed to be repeated, participants rested for 5 minutes or until they returned to their initial RPE. This ensured that participants did not partially complete the test and then immediately start again, which would introduce a secondary confounding source of fatigue. If the post-test needed to be repeated, no rest was provided because participants were expected to be fatigued from the running protocol. One person repeated the pre-test (a non-injured runner), while six people repeated the post-test—three each from the injured and non-injured groups. As a warm-up, a 30-s practice trial was completed before the pre-test.

Single-leg squat task performance was assessed using 3D motion capture. Kinematic data were recorded at 120 Hz from 10 Oqus 7 cameras (Qualisys, Gothenburg, Sweden). Thirty-three retroreflective markers were placed on the foot, shank, thigh, pelvis, and trunk (Figure 2). Only the dominant leg was instrumented. Three static trials were collected before the pre- and post-tests while participants stood upright.

#### 2.2.2 VO_2_ max testing

VO_2_ max was assessed to characterize aerobic fitness and to normalize the intensity of the high-intensity running protocol (Billat, 2001; Kaufman et al., 2006; Encarnación-Martínez et al., 2020). VO_2_ max testing was performed with a metabolic cart (HandyMET Clinic, MDI, Brazil) during an incremental treadmill protocol at a 1% fixed grade (Lourenço et al., 2011). Participants were familiarized with the treadmill and equipment and warmed up for 3 minutes at 8 km/h. The test started at an initial speed of 9 km/h and increased by 0.5 km/h every 30 s until exhaustion (Lourenço et al., 2011). Heart rate (HR) and RPE were measured every minute. The volume of oxygen consumed (VO_2_) and carbon dioxide expired (VCO_2_) were averaged within each 30 s interval and used to compute the respiratory exchange ratio (RER). When exhaustion was reached, participants completed a 5-min recovery protocol where the treadmill speed was initially decreased to 60% of the maximum running speed and further reduced by 5% each additional minute (Lourenço et al., 2011). The test was considered a valid VO_2_ max test if, at the end, two or more of the following criteria were met: RPE >9, HR > 90% of 220-age, RER >1.1, plateau of VO_2_, or the person could not continue (Quammen et al., 2012; Cortes et al., 2014). All tests were valid, that is, VO_2_ max was successfully measured in all participants. VO_2_ max was computed as the maximum value of VO_2_ observed during testing. The running speed corresponding to the VO_2_ max was used to normalize the intensity of the running protocol (Billat, 2001; Kaufman et al., 2006; Encarnación-Martínez et al., 2020). The HR corresponding to the VO_2_ max measurement was also recorded and used to verify the maintenance of high-intensity exercise during the running protocol (Pollock et al., 1998; Kaufman et al., 2006; Garber et al., 2011).

#### 2.2.3 High-intensity running protocol

After completing the single-leg squat pre-test, participants completed a high-intensity running protocol at 85% of VO_2_ max running speed on a treadmill with no inclination. The protocol was chosen according to past studies (Pollock et al., 1998; Billat, 2001; Kaufman et al., 2006; García-Pérez et al., 2014; Bellenger et al., 2019; Encarnación-Martínez et al., 2020) and was meant to simulate a high-intensity training session. The protocol duration was 60 min, which is consistent with training sessions commonly reported in recreational runners (Hespanhol Junior et al., 2013). Pilot data and past findings (Kaufman et al., 2006) suggested that high-intensity running for durations exceeding 15 min lead to the onset of extreme fatigue. Thus, the protocol consisted of four 15 min bouts of running at 85% VO_2_ max speed. Participants walked or ran at a self-selected speed for 5 min to recover between bouts. HR and RPE were assessed every 5 min to verify the intensity of the protocol during the entire test (Pollock et al., 1998; Impellizzeri et al., 2004; Kaufman et al., 2006; Garber et al., 2011; Bellenger et al., 2019). Specifically, we verified that HR was ≥ 85% HR max during the VO_2_ max test and RPE was ≥ 8 (Pollock et al., 1998; Kaufman et al., 2006; Garber et al., 2011). Participants warmed up for 5 min at a self-selected speed before the protocol started. To reduce the impact of recent training, participants refrained from exercising 2 days before the experiment.

### 2.3 Data analysis

Kinematic data were processed in Visual 3D (C-Motion, Inc., Rockville, MD, ). The three static calibration trials were averaged to compute the rotation zeroes and define the local coordinate system of each body segment. CoM position was estimated from a biomechanical model that included the dominant lower limb, pelvis, and trunk. Marker positions were not filtered prior to estimating the CoM position.

#### 2.3.1 Task performance

Performance measures for the single-leg squat task were the number of cycles, cycle time, cycle amplitude, and cycle speed. Because the hands were behind the head, the trunk had to be maintained upright, and the plantar surface of the foot had to remain in contact with the ground; knee joint flexion-extension to control the laser point produced corresponding CoM displacements. The vertical CoM position decreased when the laser position increased and *vice versa* (Supplementary Figure S1). Therefore, the vertical CoM displacements served as a surrogate for the laser point displacements. Squat cycle events were identified from the maxima and minima of the vertical CoM position. Consecutive maxima were used to define the start and end of each cycle. The minima were used to compute within-cycle displacements. The number of cycles was defined as one less than the number of maxima. The cycle time was computed as the time elapsed between consecutive maxima in seconds. The cycle amplitude was computed as the vertical CoM displacement from the first maximum defining each cycle to the minimum of the same cycle in meters. Cycle speed was defined as the ratio of cycle amplitude to time. Cycle time, amplitude, and speed were then averaged over all cycles.

#### 2.3.2 Balance dynamics

To characterize the balance dynamics before and after the running protocol, we examined transverse plane CoM displacements computed as the Euclidean distance between adjacent points. The CoM displacements were analyzed using SampEn and DFA to provide insights into the regularity and temporal correlation properties of the balance dynamics, respectively. CoM displacements were computed and analyzed in MATLAB (R2022b, update 3; MathWorks, Natick, MA, United States).

##### 2.3.2.1 Sample Entropy

SampEn, which is a model-free approach for measuring the degree of randomness in a sequence of observations (Richman and Moorman, 2000), was used to quantify the regularity of the CoM displacements. Higher SampEn values indicate more random dynamics—i.e., more information production or uncertainty about future behavior. Conversely, lower SampEn values indicate more regular dynamics—i.e., less information production or reduced uncertainty about future behavior.

SampEn is defined as the negative natural logarithm of the conditional probability that two sequences of *m* data points that are close within a tolerance *r* remain close when the sequences are increased to *m* + 1 data points. The *m* and *r* hyperparameters are respectively referred to as the template length and radius of similarity. SampEn values obtained under different conditions are most easily compared with fixed hyperparameter selections and dataset lengths, although SampEn is robust to variations in dataset length (Richman and Moorman, 2000). To select the hyperparameter values, we computed the median value of SampEn and the standard error of the SampEn estimates across all datasets, with *r* ranging from .05 to 1 in steps of .05 and *m* = 1, 2, 3, 4. We adapted the criteria suggested by Ramdani et al. (2009) to maximize the precision of the SampEn estimates and selected *m =* 1 and *r =* .25 (Supplementary Figure S1).

A modified definition includes a third hyperparameter, τ , that represents the lag between elements of the template vectors to improve estimates obtained from data that were oversampled or contain temporal correlations (Govindan et al., 2007). Here, we selected τ = 1 because this hyperparameter has minimal impact on the estimates obtained from stochastic processes containing temporal correlations (Liddy and Busa, 2023).

##### 2.3.2.2 Detrended Fluctuation Analysis

DFA is an analytical method to estimate the self-affine properties of an experimental dataset by characterizing the power law describing its diffusion over different time windows (Peng et al., 1994). The technique is based on a property of fractional Brownian motion (fBm), which is a family of nonstationary stochastic processes characterized by a single parameter, the Hurst exponent (*H*), that determines its diffusion:

$$\sigma^{2}\left(X_{i}\right)\propto n^{2H},$$ (1)

where $X_{i}$ is the processes of interest, $\sigma^{2}$ is the variance, and *n* is the time window. *H* is bounded on $]0,1[$ and characterizes the diffusion of $X_{i}$ : *H* = .5 corresponds to ordinary diffusion (i.e., Brownian motion), *H* < .5 is observed for subdiffusive processes, and *H* > .5 is observed for superdiffusive processes. A related family of stationary stochastic processes called fractional Gaussian noise (fGn) defines the increments of a fBm. Differencing a fBm produces a fGn and cumulatively summing a fGn gives its corresponding fBm, both characterized by the same *H*. For fGn, *H* determines the correlations between successive values: *H* = .5 is uncorrelated, *H* < .5 is negatively correlated (anti-persistent), and *H* > .5 is positively correlated (persistent).

DFA estimates the power law exponent of a modified diffusion equation:

$$\sigma \left(X_{i}\right)\propto n^{\alpha },$$ (2)

where $X_{i}$ and *n* are the same as Eq. 1, σ is the standard deviation, and α is the DFA exponent. α is bounded on $]0,2[$ , where α = *H* for fGn processes and α = *H* + 1 for fBm processes. α can easily be transformed from the discrete fGn/fBm model to continuum models, such as the frequency domain model relating spectral power and frequency (Delignières and Marmelat, 2012). For more details on the DFA algorithm, see Almurad and Delignières (2016).

Evenly spaced average DFA, which provides more accurate and precise estimates than the original algorithm (Almurad and Delignières, 2016), was used to estimate the standard deviation, σ , over a wide range of time windows, *n*. The window sizes ranged from 100 ms (12 data points) to 6,000 ms (720 data points). This approach produced 709 estimates of σ , which was reduced to 51 estimates evenly spaced on a logarithmic scale (Liddy and Haddad, 2018). Linear detrending was used because higher-order detrending did not affect the results.

To characterize processes described by a single power law, the DFA exponent (α) is estimated as the slope of the least-squares linear regression of log⁡σ on log⁡n . The presence of single power law is often confirmed by visual inspection of the diffusion plots, with *post hoc* justification provided in the form of the goodness of fit statistics (e.g., *R* ^2^). The main limitation of this approach is the subjective nature of that assessment. When a crossover is observed in the diffusion plot, there remains the challenge of determining the number of slope changes.

This problem has been addressed using a combination of visual inspection and selectively fitting specific regions of the diffusion plot (e.g., Collins and De Luca, 1993; Delignières et al., 2011). While visual inspection of the diffusion plots is always recommended, this qualitative check is not an objective evaluation of plausible models. Therefore, we adopted an objective, model-based approach for identifying an appropriate description of the diffusion properties of the balance dynamics, not unlike the approach adopted by Kuznetsov et al. (2013).

###### 2.3.2.2.1 Model specification

Candidate models were identified based on observations that the diffusion plots of human balance dynamics often contain one to three scaling regions (Kuznetsov et al., 2013). Thus, we consider a family of nested piecewise linear models with one to three segments. This decision was supported by visual inspection of the diffusion plots and the inclusion of a four-segment model, which was ruled out after none of the data were best described by this number of segments. For all models, y=logσ and x=log⁡n , and the residuals were assumed to be *N*(0, $\sigma^{2}$ ). The null model was a simple linear model,

$$y_{i}=\alpha_{0}+\alpha_{1}x_{i}+\varepsilon_{i},$$ (3)

where $\alpha_{0}$ is the intercept, $\alpha_{1}$ is the slope describing the power law scaling, and $\varepsilon_{i}$ are the residuals. Increasing the number of scaling regions from one to two involves the addition of two parameters to create a two-segment model,

$$y_{i}=\alpha_{0}+\alpha_{1}x_{i}+\alpha_{2}\left(x_{i}-c_{1}\right)\left(x_{i}>c_{1}\right)+\varepsilon_{i},$$ (4)

where $\alpha_{0}$ , $\alpha_{1}$ , and $\varepsilon_{i}$ have the same meaning, $\alpha_{2}$ is the change in slope from $\alpha_{1}$ , and $c_{1}$ is the crossover point where the slope changes. The term $\left(x_{i}>c_{1}\right)$ is a dummy variable, where the change in slope is only applied to the $x_{i}$ above the crossover point. The expansion to *n* segments is straightforward, requiring two additional parameters per segment, such that an *n*-segment model contains 2*n* parameters. The final model was a three-segment model,

$$y_{i}=\alpha_{0}+\alpha_{1}x_{i}+\alpha_{2}\left(x_{i}-c_{1}\right)\left(x_{i}>c_{1}\right)+\alpha_{3}\left(x_{i}-c_{2}\right)\left(x_{i}>c_{2}\right)+\varepsilon_{i},$$ (5)

where $\alpha_{3}$ and $c_{2}$ respectively represent an additional change in slope and crossover point.

###### 2.3.2.2.2 Model fitting

Model estimation was completed using constrained optimization, *fmincon,* in MATLAB, where the objective function minimized the residual sum of squares. Parameter constraints were: $\alpha_{0}\in \left[-25,0\right]$ , $\alpha_{1},\alpha_{2},\alpha_{3}\in \left[-3,3\right]$ , and $c_{1},c_{2}\in \left[2.83,6.22\right]$ . The α parameter constraints were chosen to accommodate a sufficient range of initial slopes and slope changes. The c parameter constraints prevented the crossover points from approaching either end of the log⁡n range and ensured that scaling regions contained at least four data points. The three-segment model also included an inequality constraint to enforce the minimum scaling region width: .4 < $c_{2}-c_{1}$ .

Because the multi-segment models are not guaranteed to converge to the global minimum, we used a scattershot approach to identify local minima from different initial conditions and recover the global minimum with a high degree of probability (e.g., Rohrer and Hogan, 2003; Rohrer and Hogan, 2006). Model fits were obtained from 100 initializations where parameter values were drawn from a uniform distribution spanning the intervals indicated above. Parameter estimates were partitioned using *k*-means clustering with a squared Euclidean distance metric. This procedure was done because multi-modal distributions were sometimes obtained and averaging over the 100 solutions often led to poor fit quality. Parameter estimates were averaged within each cluster and model fits compared across clusters using the residual sum of squares. The cluster with the lowest error was used to select the parameter estimates—i.e., the mean of all initializations in the cluster.

###### 2.3.2.2.3 Model selection

Models with more free parameters are more accurate but can overfit data. Model selection was completed by comparing the corrected Bayesian information criterion (BICc; McQuarrie, 1999) weights among the three candidate models to balance tradeoffs between model accuracy and complexity. BICc was computed as:

$$BICc=\ln\left(\frac{1}{N}\sum_{i=1}^{N}{\left(y_{i}-{\hat{y}}_{i}\right)}^{2}\right)+\frac{p\;\ln\left(N\right)}{N-p-2},$$ (6)

where the first term contains the mean squared error and the second term contains the number of model parameters, *p*, and the number of points in the diffusion plot, *N*. BICc weights (wBICc) were obtained following the procedures described by Wagenmakers and Farrell (2004). An easy-to-interpret measure of the relative likelihood of candidate models was obtained by taking the ratio of wBICc. For instance, if the ratio of the wBICc of model *A* to model *B* is 2.5, then model *A* is 2.5 times more likely to be the best model. If the ratio of the wBICc was greater than or equal to 2, then a model was considered better than another. If not, the simpler of the two models was chosen.

**FIGURE 3:**
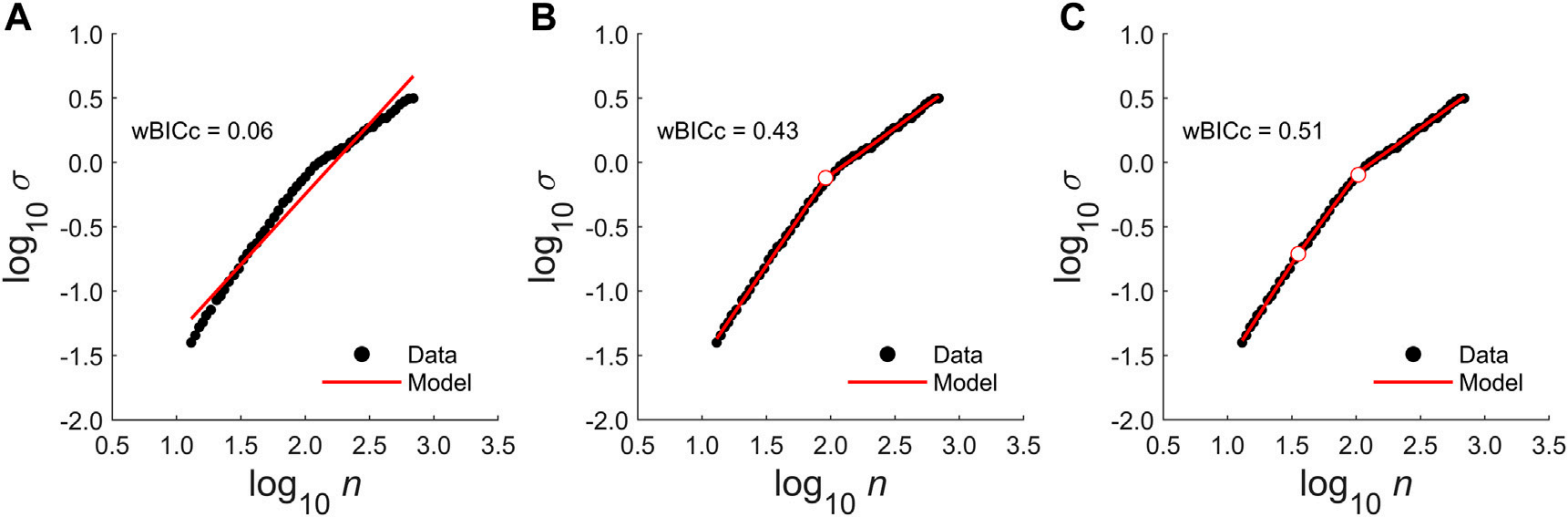
Example diffusion plot models. Each panel contains diffusion plot data from the pre-test of a single participant (black circles). Predicted values from each model are shown as red lines. **(A)** The null model was a simple linear model, which is the standard model adopted for DFA. **(B)** The two-segment model contains two scaling regions separated by a single crossover point (white circle with red outline). **(C)** The three-segment model contains three scaling regions separated by two crossover points. The weighted corrected Bayesian information criterion weights (wBICc) for each model are shown in the top left of each panel. This value indicates the likelihood, expressed as a percentage of the cumulative model weight, that a particular model is the best model. The ratios of the wBICc values provide a metric for comparing the relative likelihood of different models. For example, the two-segment model is 7.2 times more likely to be the best model than the null model. The three-segment model has the highest wBICc value, which reflects the model’s accuracy despite having more parameters. Compared to the two-segment model, it is 1.2 times more likely to the be the best model, which is below the threshold of 2. This is because the two- and three-segment models both account for large percentages of the cumulative model weight. Thus, in this instance, because there is uncertainty about which model is the best, the more parsimonious two-segment model would be selected.

The models were ranked in descending order by wBICc to select the best model. The relative likelihood of the best model (i.e., highest wBICc) to the next best model was determined. If the relative likelihood was less than 2, the simpler model was selected as the best model. The relative likelihood of the best model was then compared to the next best remaining model. This process continued until all models had been evaluated. Figure 3 shows an example diffusion plot with the three model fits and wBICc values.

### 2.4 Statistical analysis

Statistical analyses were performed in SPSS 21 (SPSS Inc., Chicago, IL, United States). To compare the characteristics of the runners assigned to injured and non-injured groups, we used two-tailed independent samples *t*-tests, and standardized effect sizes were estimated using Cohen’s *d*, which were interpreted as small (.2), medium (.5), and large (≥.8) (Cohen, 1988). Statistical significance was assessed at the α = 0.05 level. Task performance and balance dynamics outcomes were analyzed using a two-way mixed model analysis of variance (ANOVA) with Group (injured, non-injured) as a between-subjects factor, Time (pre, post) as a within-subjects factor, and participant as a random factor. Post-hoc comparisons were made using Bonferroni corrections. To examine the utility of tracking within-person changes on a relative scale, we computed the difference in the pre- and post-test values divided by the pre-test value and multiplied by 100 (i.e., 100 × (Post - Pre)/Pre). This produced a percent change score from the pre-test value, where negative values indicated that the post-test was lower and positive values indicated that the post-test was higher. This procedure was conducted for all task performance and balance dynamics outcomes. The percent change scores were analyzed using one-way ANOVAs with Group (injured, non-injured) as a between-subjects factor. In addition to comparing group means, we verified whether the group means were different from 0%. Partial eta squared (η^2^_p_) was computed to estimate effect sizes for all ANOVAs and was interpreted as small (.01), medium (.06), and large (≥.14) (Cohen, 1988).

## 3 Results

### 3.1 Injury survey

Thirteen participants (41.9%) reported sustaining an injury within 6 months of completing the experiment. All reported injuries were consistent with lower extremity overuse injuries, such as patellar and hamstring tendinopathies, piriformis and iliotibial band syndromes, and shin splints, commonly documented in runners (Hreljac, 2005; Van Gent et al., 2007; Lopes et al., 2012; Verschueren et al., 2020) and required a reduction or interruption of training or assistance from a healthcare professional. Injuries occurred between one and 5 months after completing the experimental testing, with an average of 3.2 months.

Injured runners were matched with 13 non-injured runners (Table 1). The groups were paired based on age, sex, body mass index, VO_2_ max, running experience, average pace, and average volume. The injured and non-injured groups did not differ from each other in these characteristics. Five runners reported experiencing an injury in the 6 months prior to enrolling in the study. Two of these participants were in the injured group, indicating that they were reinjured in the 6 months following testing. The other three participants were in the non-injured group, indicating that they did not pick up another injury over that same period. Five runners that did not sustain an injury were excluded from further analyses, leaving 13 in each group. Compared to the rest of the study sample, the five excluded runners were younger males (mean: 25.8 years) with greater aerobic fitness (mean VO_2_ max: 55.1 mL·min^−1^·kg^−1^) and faster average pace (mean: 4:33 min/km).

**TABLE 1:** Characteristics of the runners assigned to the injured and non-injured groups (*n* = 13 each).

	Injured	Non-injured	*p*-value	Mean difference (95% CI)	Effect size
	Mean (SD)	Mean (SD)			
Age (years)	40.5 (9.9)	39.5 (8.9)	0.81	−0.9 (−8.5, 6.7)	0.11
Sex (F/M)	6 F/7 M	5 F/8 M	0.71	−0.1 (−0.5, 0.3)	0.15
Body mass index (kg/m^2^)	23.6 (2.6)	24.5 (3.0)	0.38	1.0 (−1.3, 3.3)	0.32
VO_2_ max (ml·min^−1^·kg^−1^)	47.6 (5.0)	46.2 (5.1)	0.49	−1.4 (−5.5, 2.7)	0.28
Running experience (years)	6.1 (10.9)	6.0 (6.3)	0.97	−0.1 (−7.3, 7.1)	0.01
Average pace (min/km)	5:19 (0:36)	5:29 (0:49)	0.52	−0:11 (−0:46, 0:24)	0.24
Average volume (km/week)	42.0 (22.3)	41.4 (26.4)	0.96	0.5 (−19.3, 20.3)	0.02

SD, standard deviation; CI, confidence interval; F – female; M – male. Group means were compared using two-tailed independent samples *t*-tests with a significance level of α = .05. Mean differences and 95% confidence intervals are reported. Effect sizes were estimated using Cohen’s *d*.

The injury survey also collected information about the runner’s training routine at 6 months. The injured group reported running an average of 33.0 km/week (SD 15.08) at an average pace of 05:18 min/km (SD 00:33), while the non-injured group reported running 51.31 km/week (SD 38.0) at a pace of 05:31 min/km (SD 00:42). We analyzed the training routine data with a two-way mixed-model ANOVA to compare the initial and six-month training volume and pace (Supplementary Table S1). For training volume, there was a Time × Group interaction (*F*_1,25_ = 4.87; *p* = .037; η^2^ = .17). Bonferroni adjusted contrasts were examined within groups over time and between groups at each time. There were no statistical differences for training pace or volume (all *p* > .05), but there was a trend for decreased volume (−21.4%) in the injured group and increased volume (24.9%) in the non-injured group at 6 months. Thus, there were no changes in the recorded training variables over the six-month period.

### 3.2 Performance

Single-leg squat performance was assessed by the number of repetitions completed within 60 s, as well as spatiotemporal characteristics of the vertical CoM movements, which served as a proxy for the laser point movements. The mixed model ANOVAs did not reveal effects of Time, Group, or Time × Group for any balance performance variables (Table 2). On average, participants completed about 35 cycles during the 60-s test period, with an average cycle time of 1.78 s. On average, cycle amplitude was about 8.2 cm, and the average cycle speed was about 5 cm·s^−1^. In summary, there were no group differences in single-leg squat task performance when variables were expressed in absolute values.

**TABLE 2:** Task performance outcomes.

	Time	Injured	Non-injured	Statistical effect								
				Time			Group			Time x group		
		Mean (SD)	Mean (SD)	*F*_1,25_	*p*	η^2^_p_	*F*_1,25_	*p*	η^2^_p_	*F*_1,25_	*p*	η^2^_p_
Cycles (reps)	Pre	34.5 (7.7)	34.8 (11.2)	3.21	.09	.12	0.00	.96	.00	0.36	.56	.02
	Post	36.6 (7.4)	35.9 (13.4)									
Cycle time (s)	Pre	1.79 (0.41)	1.81 (0.42)	0.80	.38	.03	0.24	.63	.01	2.37	.14	.09
	Post	1.69 (0.42)	1.84 (0.52)									
Cycle amplitude (m)	Pre	0.077 (0.02)	0.084 (0.03)	1.57	.22	.06	0.28	.60	.01	1.18	.29	.05
	Post	0.083 (0.02)	0.084 (0.02)									
Cycle speed (ms^−1^)	Pre	0.045 (0.01)	0.052 (0.03)	3.38	.08	.12	0.16	.69	.01	2.21	.15	.08
	Post	0.052 (0.02)	0.052 (0.03)									

SD, standard deviation; reps – repetitions. Means and standard deviations are reported for pre- and post-tests for the injured and non-injured groups. The results of the two-way mixed-model ANOVAs, are reported, including F-values, *p*-values, and partial eta squared. ANOVA statistics are rounded to two decimal places.

**FIGURE 4:**
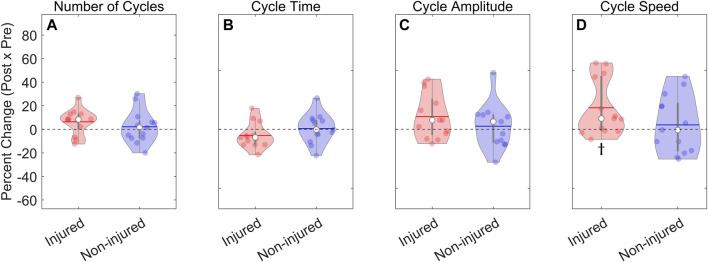
Percent change in the task performance outcomes. **(A–D)**: The within-person pre-post percent change was analyzed using a one-way ANOVA with Group (injured, non-injured) as a between-subjects factor. There was insufficient evidence of differences between the groups for all outcomes. **(A–C)** The percent change was not different from 0% in either group for the number of cycles, the cycle time, and the cycle amplitude. **(D)** However, cycle speed increased in the injured group but did not change in the non-injured group; ϯ - significantly different from 0% with *p* = .012. ● (solid dot) – within-person pre-post change; ▬○▬ – median and interquartile range; ▬ (horizontal line) – group mean.

To examine relative (within-person) changes following the high-intensity run, the within-person percent change from the pre-to post-test was computed for the task performance outcomes (Figure 4). No differences were observed in the percent change scores between the injured and non-injured groups (Table 3). The percent change for the number of cycles, cycle time, and cycle amplitude were not different from 0% in either group, indicating that the relative performance metrics were, on average, unaffected by the high-intensity run. However, the post-test cycle speed increased in the injured group (18.2%; *t*_(24)_ = 2.72; *p* = .012). Notably, the post-test cycle speed was not different from 0% in the non-injured group, and there was no difference between the groups. Thus, the only task performance outcome sensitive to the high-intensity run was the percent change in the cycle speed, which increased in the injured group. Otherwise, the injured and non-injured runners could not be discriminated based on task performance.

**TABLE 3:** Percent change for the task performance outcomes.

	Injured	Non-injured	*F*_1,24_	Mean difference (95% CI)	*p*	η^2^_p_
	Mean (SD)	Mean (SD)				
Cycles (%)	6.4 (10.8)	2.2 (14.1)	0.73	4.2 (−6.0, 14.4)	0.40	.03
Cycle Time (%)	−5.3 (10.7)	0.64 (12.5)	1.70	−6.0 (−15.4, 3.5)	0.21	.07
Amplitude (%)	10.8 (19.1)	2.7 (18.9)	1.20	8.15 (−7.2, 23.5)	0.29	.05
Speed (%)	18.3 (23.9)^a^	3.8 (24.4)	2.32	14.4 (−5.1, 34.0)	0.14	.09

SD, standard deviation; CI, confidence interval.

^a^ – significantly different from 0% (*t*_(24)_ = 2.72, *p* = .01).

Percent change was measured as the difference between the pre- and post-test values divided by the pre-test values for each participant. The results of the one-way ANOVAs, including F-values, *p*-values, and partial eta squared comparing the percent change between the injured and non-injured groups are reported. ANOVA statistics are rounded to two decimal places.

### 3.3 Balance dynamics

**FIGURE 5:**
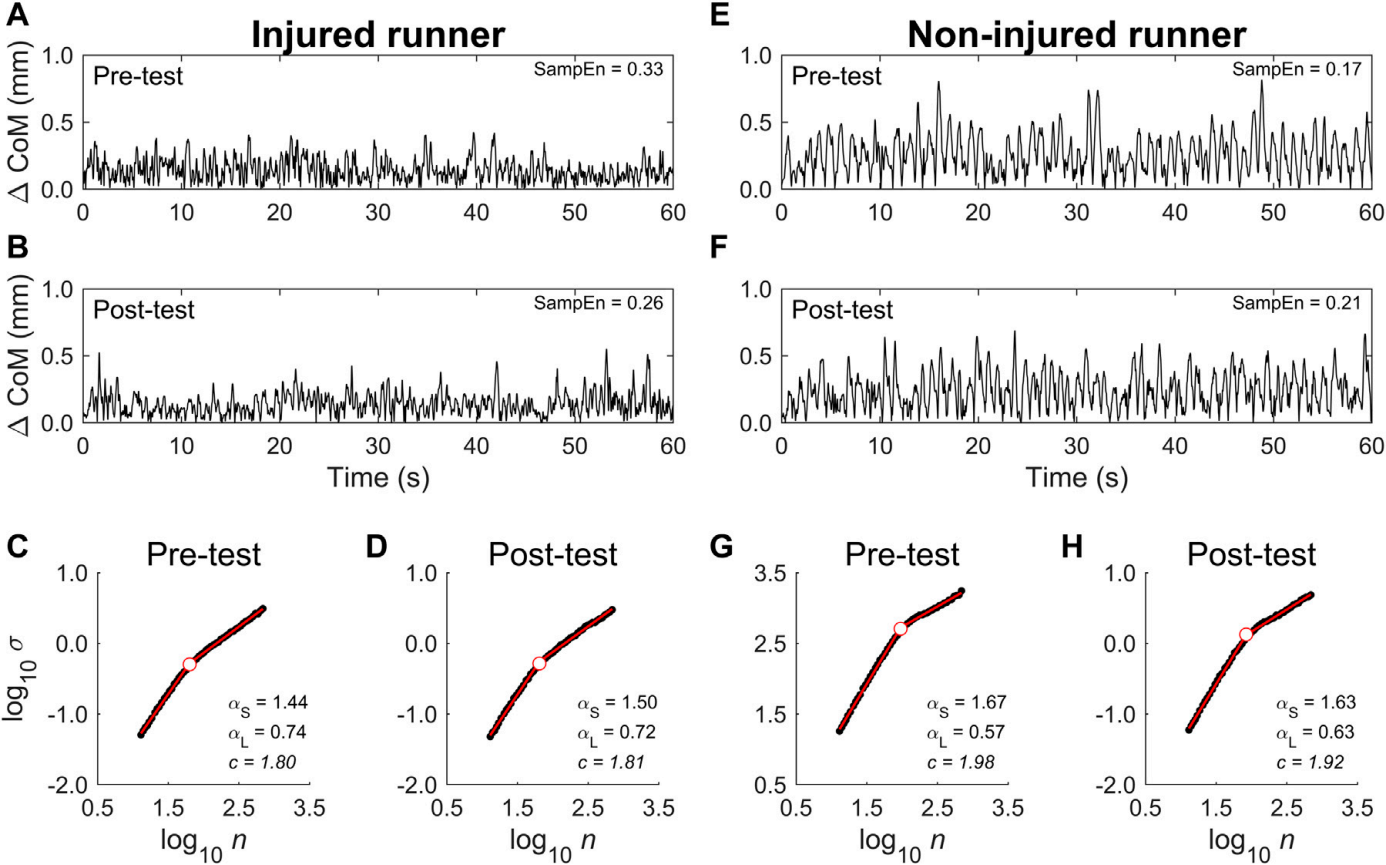
Example CoM displacement time series and diffusion plots from a representative injured and non-injured runner. Data from the injured runner is displayed in panels **(A–D)**, while data from the non-injured runner is shown in **(E–H)**. CoM displacements from the 60 s single-leg squat task for the pre-test [**(A)** injured, **(E)** non-injured] and post-test [**(B)** injured, **(F)** non-injured] show short-term drifts and long-term stationarity, consistent with past work. From visual inspection, the non-injured runner displays more regular dynamics characterized by intermittent periods of limit-cycle behavior compared to the more stochastic, less regular injured runner. This was confirmed by examining SampEn, which is displayed in the upper right corner of **(A,B,E,F)**. The corresponding diffusion plots for the pre-test [**(C)** injured, **(G)** non-injured] and post-test [**(D**) injured, **(H)** non-injured] confirm the presence of crossovers. The two-segment model contains a short-term scaling region and a long-term scaling region before and after the crossover point, respectively. The short-term exponents ( $\alpha_{S}$ ) were greater than 1 and close to 1.5, indicating nonstationary diffusion characterized by nearly uncorrelated increments in the CoM displacements up to the crossover point ( c ) at about 500–750 ms. This is not unexpected because the CoM displacements per unit time (i.e., speed) was bounded from repeatedly squatting up and down. The long-term exponents ( $\alpha_{L}$ ) following the crossover point were below 1, indicating stationarity, but were characterized by persistent fluctuations, such that the bounding of speed is not strict, but exhibits local drifts.

Balance dynamics during the single-leg squat test were assessed by SampEn and a two-segment model DFA of CoM displacements in the transverse plane. SampEn of the transverse plane CoM displacements was used to quantify regularity, while DFA quantified temporal correlations. Example CoM displacement time series and the associated diffusion plots from an injured and non-injured runner are displayed in Figure 5.

**FIGURE 6:**
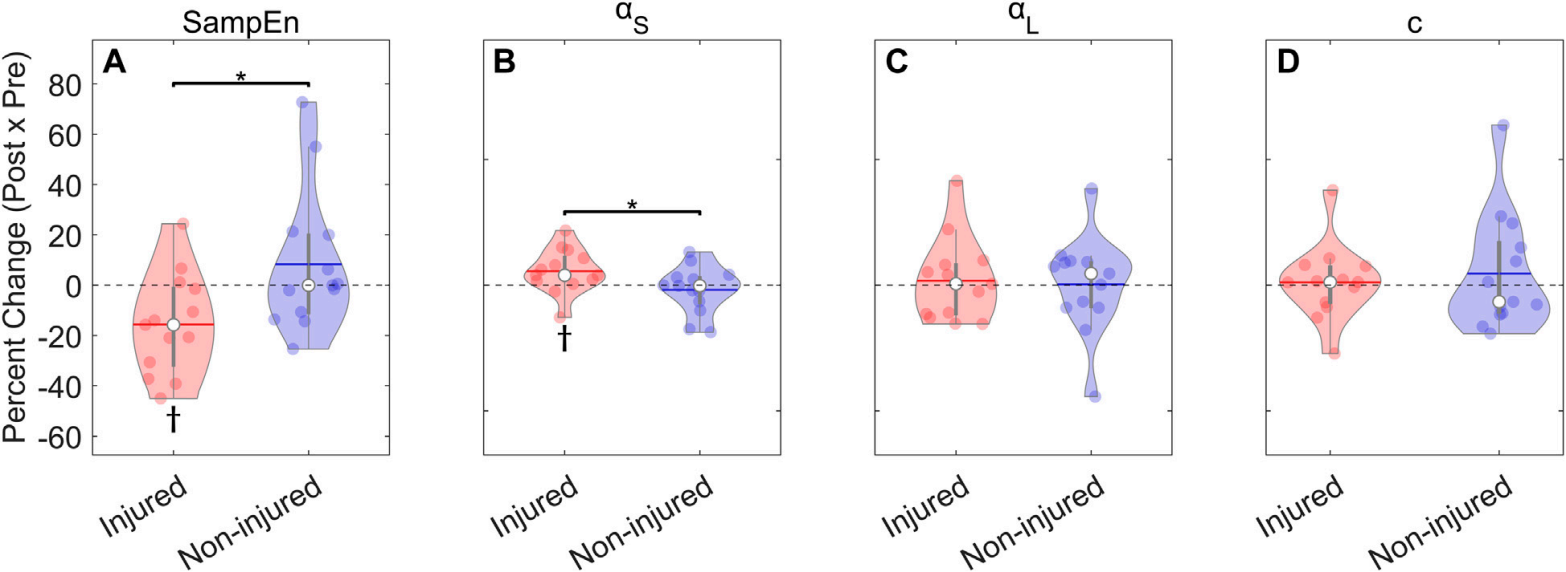
Percent change from post to pre-test in balance dynamics: Sample Entropy (SampEn) and Detrended Fluctuation Analysis (DFA). The injured group decreased SampEn and increased Short-term α after high-intensity run, while the non-injured group did not change. $\alpha_{S}$ – Short-term scaling exponent; $\alpha_{L}$ – Long-term scaling exponent; *c* – Crossover point; * - siginificant difference between groups (*p* < 0.05); ϯ - significantly different from 0% (*p* < 0.05); ● (solid dots) – individual results; ▬○▬ – median and interquartile range; ▬ (horizontal lines) – group mean.

SampEn estimates were generally low, with a range of .13–.59, indicating a high degree of regularity (Table 4). The two-way mixed-model ANOVA showed a significant Time **×** Group interaction, which indicated increased regularity (i.e., decreased SampEn) in the injured group from pre-to post-test, while no other comparisons were statistically significant. This indicated that 1) the regularity of the CoM displacements was not different between the injured and non-injured groups in the pre-test, 2) regularity did not differ following the high-intensity run in the non-injured group, and 3) decreased regularity in the injured group following the high-intensity run. The percent change scores followed the same pattern, indicating that only the injured group changed after the high-intensity run with an average reduction in SampEn of 15.6% (Figure 6A; Table 5, SampEn). In summary, the balance dynamics of runners who reported experiencing an injury in the 6 months following assessment became more regular after completing the high-intensity run while those who did not, on average, showed no change.

**TABLE 4:** Balance dynamics.

	Time	Injured	Non-injured	Statistical effect								
				Time			Group			Time × group		
		Mean (SD)	Mean (SD)	*F*_1,25_	*p*	η^2^_p_	*F*_1,25_	*p*	η^2^_p_	*F*_1,25_	*p*	η^2^_p_
SampEn	Pre	0.29 (0.07)	0.28 (0.08)	1.28	.27	.05	0.33	.57	.01	7.04	.014*^a^	.23
	Post	0.23 (0.04)	0.29 (0.12)									
$\alpha_{S}$	Pre	1.46 (0.14)	1.50 (0.11)	0.76	.39	.03	0.02	.89	.00	4.02	.056	.14
	Post	1.53 (0.09)	1.47 (0.18)									
$\alpha_{L}$	Pre	0.69 (0.11)	0.66 (0.07)	0.01	.92	.00	1.07	.31	.04	0.33	.57	.00
	Post	0.70 (0.12)	0.65 (0.12)									
*C*	Pre	0.62 (0.09)	0.64 (0.14)	0.25	.62	.01	0.36	.56	.02	0.034	.85	.01
	Post	0.63 (0.14)	0.66 (0.14)									

SD, standard deviation; * - statistical significance (*p* < 0.05).

^a^ – Post-hoc with Bonferroni adjustment revealed a difference in the injured group (post < pre; *F*_1,25_ = 7.17; *p* = .01; η2 = .23). $\alpha_{S}$ – Short-term DFA exponent; $\alpha_{L}$ – Long-term DFA exponent; *c* – crossover point.

Sample Entropy (SampEn) and Detrended Fluctuation Analysis (DFA) outcomes were analyzed with two-way mixed-model ANOVAs, with Time and Group as fixed factors and participant as a random factor. Means and standard deviations are reported for pre- and post-tests and the injured and non-injured groups. The results of the two-way mixed-model ANOVAs, are also reported, including F-values, *p*-values, and partial eta squared. ANOVA statistics are rounded to two decimal places.

**TABLE 5:** Percent change in balance dynamics outcomes.

	Injured	Non-injured	*F*_1,24_	Mean difference (95% CI)	*p*	η^2^_p_
	Mean (SD)	Mean (SD)				
SampEn (%)	−15.6 (19.9)^a^	8.3 (28.1)	6.29	−24.0 (−43.7, −4.3)	.02*	.21
$\alpha_{S}$ (%)	5.5 (8.7)^b^	−1.9 (9.4)	4.44	7.5 (0.2, 14.8)	.04*	.16
$\alpha_{L}$ (%)	1.8 (16.5)	0.3 (19.2)	0.05	1.5 (−13.0, 16.0)	.83	.00
*c* (%)	1.1 (15.0)	4.6 (23.3)	0.22	−3.6 (−19.4, 12.3)	.65	.01

SD, standard deviation; * - statistical significance (*p* < .05).

^a^ – significantly different from 0% (*t*_(24)_ = −2.31, *p* = 0.03).

^b^ – significantly different from 0% (*t*_(24)_ = 2.21, *p* = 0.04); $\alpha_{S}$ – Short-term DFA exponent; $\alpha_{L}$ – Long-term DFA exponent; *c* – crossover point.

The within-person percent change in the Sample Entropy (SampEn) and Detrended Fluctuation Analysis (DFA) outcomes was computed as the difference between the pre- and post-test values divided by the pre-test values. The results of the one-way ANOVAs, including F-values, *p*-values, and partial eta squared comparing the percent change between the injured and non-injured groups are reported. ANOVA statistics are rounded to two decimal places.

DFA was calculated with an objective approach to investigate the appropriate diffusion properties of the balance dynamics. Because the diffusion plots of postural displacements often contain one or more crossovers, we compared a family of nested, piecewise linear models with one to three segments. In total, we examined 52 CoM displacement time series (2 groups × 13 participants × 2 time points). A substantial majority of the data (51, ∼98%) were best described by the two-segment model, with the three-segment model only selected once. Thus, we adopted the two-segment model to characterize the scaling behavior of the CoM displacements.

The two-segment model contains four parameters (Eq. 2), three of which are relevant to describing the balance dynamics: $\alpha_{1}$ , $\alpha_{2}$ , and $c_{1}$ . The scaling exponent $\alpha_{1}$ represents the initial slope of the diffusion plot from the shortest timescales to the crossover point ( $c_{1}$ ), while $\alpha_{2}$ represents the change in slope after $c_{1}$ . For simplicity, we will refer to the short-term scaling exponent as $\alpha_{S}=\alpha_{1}$ , the long-term scaling exponent as $\alpha_{L}=\alpha_{1}+\alpha_{2}$ , and the crossover point as $c=c_{1}$ , which was converted to units of seconds rather than log *n*. Crucially, short- and long-term denote the relation to the crossover point, not necessarily to the physiological processes involved in balance control.

The two-way mixed-model ANOVAs results from DFA parameters— $\alpha_{S}$ , $\alpha_{L}$ , and c —are shown in Table 4. For $\alpha_{S}$ , there were no significant effects of Time or Group, while the Time **×** Group interaction indicated a large effect but did not reach statistical significance (*p* = .056). For both $\alpha_{L}$ and c , there were no effects of Time or Group, and no interaction. On average, the short-term CoM displacements were nonstationary ( $\alpha_{S}$ > 1) with nearly uncorrelated increments. The crossover, *c*, occurred around 0.64 s and was unchanged following the high-intensity run, indicating that the bounding of the CoM displacements remained the same. The long-term CoM displacements were, on average, stationary ( $\alpha_{L}$ < 1) and characterized by persistent correlations ( $\alpha_{L}$ > .5).

By contrast, one-way ANOVAs examining the percent change scores for the DFA parameters revealed several group differences. Specifically, $\alpha_{S}$ increased in the injured group following the high-intensity run (*t*_(24)_ = 2.21, *p* = 0.04), indicating more diffusive short-term CoM dynamics, whereas it was unchanged in the non-injured group (*t*_(24)_ = −0.76, *p* = 0.46). Moreover, there was also evidence of group differences related to pre-post percent change (Figure 6B; Table 5). Neither $\alpha_{L}$ or c changed in either group following the high-intensity run and there was insufficient evidence of group differences (Figures 6C, D; Table 5). In summary, the injured group was more affected by fatigue and showed more diffusive short-term CoM displacements following the high-intensity run, compared to the non-injured group.

## 4 Discussion

This prospective study investigated changes in balance dynamics during a single-leg squat task following a high-intensity run in recreational runners who did or did not become injured in the 6 months following assessment. As predicted, task performance outcomes, which included the number of squats completed and the average cycle time, amplitude, and speed, were not different in the injured and non-injured runners and were not affected by fatigue (Prediction 1). The predictions related to balance dynamics, which included measures of regularity (SampEn) and temporal correlations (DFA), were partially supported. Injured and non-injured runners did not exhibit differences in balance dynamics before the high-intensity run (Prediction 2). Counter to Predictions 3 and 4, fatigue-induced changes in regularity (lower SampEn) and temporal correlations (greater $\alpha_{S}$ ) were only observed in the injured group, contrary to expectations. The pre-post percent change in measures of balance dynamics indicated increased regularity and more persistent short-term correlation structure in injured runners in response to fatigue, but no changes in non-injured runners. Thus, the non-injured runners demonstrated some degree of fatigue-resistance in balance dynamics, which was not predicted. Also, as predicted, there were no group differences or fatigue-related changes in the long-term correlation structure or crossover point, which is consistent with the lack of task-level changes (Prediction 5). Finally, we developed an objective, model-based approach for characterizing crossovers in balance dynamics, with implications for identifying and quantifying crossover phenomena in human behavior.

Changes to balance dynamics following high-intensity running were observed in recreational runners that became injured in the next 6 months. Specifically, the injured group demonstrated a 15.6% reduction in SampEn and a 5.6% increase in the short-term scaling exponent. Increased regularity and DFA scaling exponent in movement dynamics have been previously observed in response to fatigue (Pethick et al., 2015; 2016; Vázquez et al., 2016) and in individuals with injury or injury history (Georgoulis et al., 2006; Tochigi et al., 2012; Quatman-Yates et al., 2015; Terada et al., 2015; Quirino et al., 2021). For example, injured individuals showed greater regularity (lower SampEn) of balance dynamics during relaxing standing, compared to healthy controls (Quatman-Yates et al., 2015). These results are commonly interpreted as a reduced capacity to meet task demands, which may be associated with a reduction in adaptability following fatigue-inducing exercise (Vázquez et al., 2016; Balagué et al., 2020). For instance, Montull et al. (2020) reported that more persistent ankle acceleration dynamics was associated to worse self-reported performance in slackline walking, which they interpreted as an indication of less adaptable behavior. Only the injured runners were affected by the fatigue protocol. The non-injured runners did not demonstrate significant changes to their balance dynamics after the high-intensity run, which may indicate a fatigue resistance or the ability to mitigate the deleterious consequences of exercise-induced fatigue (Monjo et al., 2015). Therefore, these findings provide support for the proposal of Fonseca et al. (2020) that the capacity to withstand perturbations from training and fatigue is fundamental to reducing injury occurrence.

Notably, the short-term scaling exponents indicated that the CoM displacements were nonstationary (α > 1) and characterized by ordinary diffusion (α ≈ 1.5), such that variance increased proportionally with time. Past studies have commonly reported stationary and positively correlated (.5 < *α* < 1) postural displacements (e.g., Collins and De Luca, 1993; Delignières et al., 2011). But, nonstationary subdiffusive (1 < *α* < 1.5) dynamics (Kodama et al., 2022) and superdiffusive (α > 1.5) dynamics (van den Hoorn et al., 2018) have also been reported during single-leg and relaxed standing, respectively. Short-term persistence reflects drifts in postural displacements, with suggestions indicating that these are related to open-loop control (Collins and De Luca, 1993), exploratory behavior (Riley et al., 1997a), threshold-based control (Delignières et al., 2011), or inertial movements (Liebovitch and Yang, 1997). Our data cannot distinguish between these possibilities—for instance, it would be challenging to disentangle exploratory and inertial contributions. Increased $\alpha_{S}$ in the injured group reflects greater diffusion of the CoM displacements per unit time, which is consistent with the increase in average cycle speed, which was also observed. This corresponded to the more regular CoM displacements captured by SampEn, which aligns with expectations for how these variables should co-vary for nonstationary stochastic processes (Liddy and Busa, 2023). More importantly, the injured group was, on average, distinguished by the changes in SampEn and short-term correlation structure, which seem to provide meaningful indicator of reduced capacity to recover from fatigue-inducing exercise and may be associated with a greater risk of future injury.

Examining within-person percent changes to balance dynamics in response to the fatigue protocol better distinguished the injured and non-injured groups compared to the mixed-model approach, which examined the absolute values. Lower SampEn was seen in the injured group following the high-intensity run, whereas the increase in the short-term exponent did not reach statistical significance, despite the large effect size for the Group × Time interaction (η^2^_p_ = .14). But, overall, there was no evidence of group differences. This suggests that comparing within-person percent changes in balance dynamics over time may provide more sensitive metrics for tracking clinically relevant outcomes, such as injury occurrence in recreational runners, than examining absolute values alone. Evaluating within-person percent change may also be beneficial for comparing results across different studies and populations or establishing clinically meaningful change. The reason is that comparing just the absolute values of entropy and DFA is challenging due to differences in task, dataset length, data cleaning, and hyperparameter selection, which can impact results (Ducharme and Van Emmerik, 2018; Liddy and Busa, 2023). However, it is still imperative to report the absolute values because they contain descriptive information about underlying processes.

As predicted, task performance did not differ between the injured and non-injured groups before or after the high-intensity run. Our results are congruent with previous observations that fatigue did not impair performance on single-leg tests (Zech et al., 2012; Heil and Büsch, 2022). Although performance (i.e., number of repetitions, cycle time, and amplitude)and physiological (e.g., VO_2_ max) variables are commonly considered markers of fatigue or fitness (Balagué et al., 2020; Garcia-Retortillo and Ivanov, 2022), they did not discriminate injured from non-injured runners following high-intensity exercise. However, the injured group increased cycle speed following the high-intensity run, while the non-injured group did not. Fatigue-related increases in postural sway velocity are commonly assumed to be maladaptive (Corbeil et al., 2003; Lin et al., 2009; Zech et al., 2012). But, task performance can be maintained even when movement patterns change following fatigue (Gates and Dingwell, 2008). Thus, we interpret the increased cycle speed, regularity, and short-term correlation structure in the injured group as compensatory adaptations to maintain task performance in response to fatigue. Moreover, these findings suggest that examining task performance in isolation can be insufficient to detect changes in movement that reflect future injury occurrence.

Similarly, as predicted, the long-term correlation structure was not impacted by the high-intensity run and did not differ between the injured and non-injured groups. In this study, the long-term region encompassed multiple cycles (from .33 to 3.33 cycles), and as a result was more reflective of task-level dynamics. The long-term region was, on average, characterized by stationary persistent correlations (.5 < *α* < 1). Stationary, persistent correlations have been observed in the long-term region during relaxed standing (van den Hoorn et al., 2018). But, long-term scaling has consistently been described by stationary, anti-persistent correlations (α < .5) during quiet standing (Collins and De Luca, 1993; Collins and De Luca, 1995a; Collins and De Luca, 1995b; Delignières et al., 2011). Such dynamics are consistent with an intermittent control strategy that seeks to reverse postural displacements in an event-driven manner (Gawthrop et al., 2011), such as when a velocity threshold is crossed (Delignières et al., 2011). By contrast, in the single-leg squat task, the CoM displacements were less tightly regulated—i.e., the statistical tendency to reverse direction was not observed—suggesting a modified control strategy compared to quiet standing. Therefore, the long-term correlation structure seems to reflect the degree of corrective control required to meet concurrent demands related to postural stability and suprapostural task performance.

Another expectation was that the crossover point, which indicates the timescale marking the change in correlation structure, would not be affected by fatigue or differ between the groups. This expectation was related to the prediction of no changes in task performance, as well as the observation that the crossover point can reflect periodic trends (Hu et al., 2001). Squatting induces quasiperiodic transverse plane movements because the CoM tends to shift forward and ipsilateral during the eccentric phase and backward and contralateral during the concentric phase. Crossovers can reflect statistical artifacts, which can be filtered out to recover a single-scaling region (e.g., Anastas et al., 2011). However, the single-leg squat task results in quasiperiodic CoM movements that are an inextricable part of the movement dynamics. Thus, changes in crossover points would only be expected when accompanied by differences in the movement frequency.

Past studies examining crossovers in balance dynamics have relied mainly on visual inspection to identify the number and location of the short- and long-term regions (e.g., Collins and De Luca, 1993; Delignières et al., 2011). Moreover, the intersection of the lines fitted to the two regions has been used to identify the crossover point (e.g., Collins and De Luca, 1993) rather than including it as an additional model parameter. To mitigate these concerns, we adopted an objective, model-based approach that expands on the work of Kuznetsov et al. (2013). The guiding principle of our approach is to identify a parsimonious solution that balances model accuracy with simplicity while avoiding *a priori* assumptions about how many scaling regions are present. The default model was a simple linear model, which represents a single scaling region, consistent with the discrete fGn/fBm model or the continuum 1/ $f^{-\beta }$ model. Multi-segment piecewise linear models were then evaluated against this null hypothesis using model weights to minimize selection biases and penalize overly complex models. Crucially, this approach can easily be translated to aid in the identification and quantification of crossover phenomena in many types of neurophysiological, biomechanical, behavioral, and even psychological measures.

## 5 Limitations

This study has multiple limitations. First, retrospective surveys rely on the ability to recall past events and may not always provide reliable information (Schwarz, 1999). Despite this drawback, retrospective surveys are useful tools for documenting injuries (e.g., Jenkins et al., 2002; Gabbe et al., 2003). Because athletes can have trouble recalling the specific nature or extent of their injuries (Gabbe et al., 2003), future work may benefit from more frequent check-ins to mitigate time-dependent memory effects (Jenkins et al., 2002) and identify whether changes balance dynamics are associated with injury onset.

Second, considering the multifactorial nature of sports injuries, many factors may have contributed to the reported injuries. However, both groups were free of injury at the initial assessment and had similar characteristics commonly associated with injury risk, such as previous injury history and training variables. Moreover, there was no association between training variables or aerobic fitness and task performance or balance dynamics, reaffirming that these factors did not influence the reported findings. In the six-months after assessment, the injured group decreased their weekly training volume by 21.4%, while the non-injured group increased their weekly training volume by 23.9% relative to the initial assessment. The decline in training volume in the injured group was not unexpected given the definition of running injury adopted in this study. But, more importantly, these data rule out the possibility that the reported injuries were due to an increase in training volume. While the injury survey inquired about all-cause injuries, all reported injuries were overuse injuries commonly observed in runners (Hreljac, 2005; Van Gent et al., 2007; Lopes et al., 2012; Verschueren et al., 2020; Willwacher et al., 2022). Because overuse injuries refer to musculoskeletal insults resulting from repeated stress over time (Hreljac, 2005), they are mainly related to a lower capacity to adapt to the stresses imposed by training and other physical activities (Fonseca et al., 2020). Therefore, this promising approach may provide insights into how the ability to respond to fatiguing exercise may reveal impending overuse injury. Still, the inclusion of physiological variables (e.g., intra- and inter-muscular coordination) that are affected by fatigue and related to injury were not assessed here and should be considered in future studies using tools and concepts from the field of network physiology (Bashan et al., 2012; Balagué et al., 2020).

Third, the study sample was small (*N* = 31), which reduces statistical power and the precision of effect size estimates, and limits the generalizability (Hackshaw, 2008). However, based on the strong evidence of changes to balance dynamics in the injured group, as indicated by large effect sizes, the experimental approach adopted here should be investigated in larger samples. Moreover, this study provides preliminary evidence of group-level differences in the balance dynamics of recreational runners that did and did not experience an injury over half a year. While the sensitivity and specificity of this specific protocol for predicting running-related injuries remain to be seen, we believe our general approach will contribute to a better understanding of future injury occurrence and the development of preventive strategies for overuse running-related injuries.

## 6 Conclusion

Recreational runners that reported an injury in the 6 months following assessment demonstrated changes in balance dynamics during a prolonged single-leg squat task when fatigued compared to a matched group of runners who did not become injured. Specifically, runners who sustained an injury demonstrated more regular and diffusive short-term CoM displacements following a high-intensity running protocol. Additionally, single-leg squat task performance measured by common spatial and temporal outcomes was not affected by fatigue and did not differ by future injury status in either group. Thus, examining balance dynamics during a single-leg squat task following a high-intensity training distinguished the injured and non-injured groups of runners, whereas performance variables in isolation were insufficient to identify fatigue-related changes that may be indicative of future injury occurrence. We also demonstrate the promise of tracking within individual relative changes (i.e., percent change) in balance dynamics over time for evaluating the effect of fatigue and its association with future injury occurrence. Finally, conducting functional balance assessments, such as the 60 s single-leg squat test, before and after training or exercises may provide valuable early information for identifying reduced adaptative capacity of recreational runners that may ultimately lead to injury.

## Data Availability

The data, analysis routines, and results presented in this study are openly available at https://doi.org/10.5281/zenodo.10070118.

## References

[B1] Almurad Z. M. H. Delignières D. (2016). Evenly spacing in detrended fluctuation analysis. Phys. A Stat. Mech. its Appl. 451, 63–69. 10.1016/j.physa.2015.12.155

[B2] Anastas J. R. Stephen D. G. Dixon J. A. (2011). The scaling behavior of hand motions reveals self-organization during an executive function task. Phys. A Stat. Mech. its Appl. 390, 1539–1545. 10.1016/j.physa.2010.11.038

[B3] Balagué N. Hristovski R. Almarcha M. Garcia-Retortillo S. Ivanov P. C. (2020). Network physiology of exercise: vision and perspectives. Front. Physiol. 11, 611550. 10.3389/fphys.2020.611550 33362584 PMC7759565

[B4] Bashan A. Bartsch R. P. Kantelhardt J. W. Havlin S. Ivanov P. C. (2012). Network physiology reveals relations between network topology and physiological function. Nat. Commun. 3, 702. 10.1038/ncomms1705 22426223 PMC3518900

[B5] Bellenger C. R. Arnold J. B. Buckley J. D. Thewlis D. Fuller J. T. (2019). Detrended fluctuation analysis detects altered coordination of running gait in athletes following a heavy period of training. J. Sci. Med. Sport 22, 294–299. 10.1016/j.jsams.2018.09.002 30220574

[B6] Billat L. V. (2001). Interval training for performance: a scientific and empirical practice. Special recommendations for middle- and long-distance running. Part I: aerobic interval training. Sport. Med. 31, 13–31. 10.2165/00007256-200131010-00002 11219499

[B7] Bittencourt N. F. N. Meeuwisse W. H. Mendonça L. D. Nettel-Aguirre A. Ocarino J. M. Fonseca S. T. (2016). Complex systems approach for sports injuries: moving from risk factor identification to injury pattern recognition - narrative review and new concept. Br. J. Sports Med. 50, 1309–1314. 10.1136/bjsports-2015-095850 27445362

[B8] Bittencourt N. F. N. Ocarino J. M. Mendonça L. D. Hewett T. E. Fonseca S. T. (2012). Foot and hip contributions to high frontal plane knee projection angle in athletes: a classification and regression tree approach. J. Orthop. Sport. Phys. Ther. 42, 996–1004. 10.2519/jospt.2012.4041 22990391

[B9] Borg G. (1998). Borg’s perceived exertion and pain scales. Champaign: Human Kinetics.

[B10] Buist I. Bredeweg S. W. Bessem B. Van Mechelen W. Lemmink K. A. P. M. Diercks R. L. (2010). Incidence and risk factors of running-related injuries during preparation for a 4-mile recreational running event. Br. J. Sports Med. 44, 598–604. 10.1136/bjsm.2007.044677 18487252

[B11] Burnham J. M. Yonz M. C. Robertson K. E. McKinley R. Wilson B. R. Johnson D. L. (2016). Relationship of hip and trunk muscle function with single leg step-down performance: implications for return to play screening and rehabilitation. Phys. Ther. Sport 22, 66–73. 10.1016/j.ptsp.2016.05.007 27592407

[B12] Busa M. A. Van Emmerik R. E. A. (2016). Multiscale entropy: a tool for understanding the complexity of postural control. J. Sport Heal. Sci. 5, 44–51. 10.1016/j.jshs.2016.01.018 PMC6188573 30356502

[B13] Cardoso V. A. Resende R. A. Aquino C. F. Andrade A. G. P. Silva P. L. P. Amaral G. M. (2021). A novel single-leg squat test with speed and accuracy requirements: reliability and validity in anterior cruciate ligament reconstructed individuals. Knee 29, 150–159. 10.1016/j.knee.2021.01.031 33636564

[B14] Carpenter M. G. Murnaghan C. D. Inglis J. T. (2010). Shifting the balance: evidence of an exploratory role for postural sway. Neuroscience 171, 196–204. 10.1016/j.neuroscience.2010.08.030 20800663

[B15] Ceyssens L. Vanelderen R. Barton C. Malliaras P. Dingenen B. (2019). Biomechanical risk factors associated with running-related injuries: a systematic review. Sport. Med. 49, 1095–1115. 10.1007/s40279-019-01110-z 31028658

[B16] Clansey A. C. Hanlon M. Wallace E. S. Lake M. J. (2012). Effects of fatigue on running mechanics associated with tibial stress fracture risk. Med. Sci. Sports Exerc. 44, 1917–1923. 10.1249/MSS.0b013e318259480d 22525776

[B17] Cohen J. (1988). Statistical power analysis for the behavioral sciences. 2nd edition. New York: Lawrence Erlbaum Associates.

[B18] Collins J. J. De Luca C. J. (1993). Open-loop and closed-loop control of posture: a random-walk analysis of center-of-pressure trajectories. Exp. Brain Res. 95, 308–318. 10.1007/BF00229788 8224055

[B19] Collins J. J. De Luca C. J. (1994). Random walking during quiet standing. Am. Phys. Soc. 73, 764–767. 10.1103/PhysRevLett.73.764 10057531

[B20] Collins J. J. De Luca C. J. (1995a). The effects of visual input on open-loop and closed-loop postural control mechanisms. Exp. Brain Res. 103, 151–163. 10.1007/BF00241972 7615030

[B21] Collins J. J. De Luca C. J. (1995b). Upright, correlated random walks: a statistical-biomechanics approach to the human postural control system. Chaos 5, 57–63. 10.1063/1.166086 12780156

[B22] Corbeil P. Blouin J. S. Bégin F. Nougier V. Teasdale N. (2003). Perturbation of the postural control system induced by muscular fatigue. Gait Posture 18, 92–100. 10.1016/S0966-6362(02)00198-4 14654212

[B23] Cortes N. Onate J. Morrison S. (2014). Differential effects of fatigue on movement variability. Gait Posture 39, 888–893. 10.1016/j.gaitpost.2013.11.020 24370441 PMC3960345

[B24] Delignières D. Marmelat V. (2012). Fractal fluctuations and complexity: current debates and future challenges. Crit. Rev. Biomed. Eng. 40, 485–500. 10.1615/CritRevBiomedEng.2013006727 23356693

[B25] Delignières D. Torre K. Bernard P. L. (2011). Transition from persistent to anti-persistent correlations in postural sway indicates velocity-based control. PLoS Comput. Biol. 7, e1001089. 10.1371/journal.pcbi.1001089 21390333 PMC3044760

[B26] Donker S. F. Ledebt A. Roerdink M. Savelsbergh G. J. P. Beek P. J. (2008). Children with cerebral palsy exhibit greater and more regular postural sway than typically developing children. Exp. Brain Res. 184, 363–370. 10.1007/s00221-007-1105-y 17909773 PMC2137946

[B27] Duarte M. Zatsiorsky V. M. (2000). On the fractal properties of natural human standing. Neurosci. Lett. 283, 173–176. 10.1016/S0304-3940(00)00960-5 10754215

[B28] Ducharme S. W. Van Emmerik R. E. A. (2018). Fractal dynamics, variability, and coordination in human locomotion. Kinesiol. Rev. 7, 26–35. 10.1123/kr.2017-0054

[B29] Encarnación-Martínez A. Sanchis-Sanchis R. Pérez-Soriano P. García-Gallart A. (2020). Relationship between muscular extensibility, strength and stability and the transmission of impacts during fatigued running. Sport. Biomech. 00, 1364–1380. 10.1080/14763141.2020.1797863 32835623

[B30] Fonseca S. T. Souza T. R. Verhagen E. van Emmerik R. Bittencourt N. F. N. Mendonça L. D. M. (2020). Sports injury forecasting and complexity: a synergetic approach. Sport. Med. 50, 1757–1770. 10.1007/s40279-020-01326-4 32757162

[B31] Gabbe B. J. Finch C. F. Bennell K. L. Wajswelner H. Fukuda J. (2003). Whole body muscle hypertrophy from resistance training: distribution and total mass. Br. J. Sports Med. 37, 543–545. 10.1136/bjsm.37.6.543 14665598 PMC1724694

[B32] Garber C. E. Blissmer B. Deschenes M. R. Franklin B. A. Lamonte M. J. Lee I. M. (2011). American College of Sports Medicine position stand. Quantity and quality of exercise for developing and maintaining cardiorespiratory, musculoskeletal, and neuromotor fitness in apparently healthy adults: guidance for prescribing exercise. Med. Sci. Sports Exerc. 43, 1334–1359. 10.1249/MSS.0b013e318213fefb 21694556

[B33] García-Pérez J. A. Pérez-Soriano P. Llana Belloch S. Lucas-Cuevas Á. G. Sánchez-Zuriaga D. (2014). Effects of treadmill running and fatigue on impact acceleration in distance running. Sport. Biomech. 13, 259–266. 10.1080/14763141.2014.909527 25325770

[B34] Garcia-Retortillo S. Ivanov P. C. (2022). Inter-muscular networks of synchronous muscle fiber activation. Front. Netw. Physiol. 2, 1059793–1059828. 10.3389/fnetp.2022.1059793 36926057 PMC10012969

[B35] Gates D. H. Dingwell J. B. (2008). The effects of neuromuscular fatigue on task performance during repetitive goal-directed movements. Exp. Brain Res. 187, 573–585. 10.1007/s00221-008-1326-8 18327575 PMC2825378

[B36] Gawthrop P. Loram I. Lakie M. Gollee H. (2011). Intermittent control: a computational theory of human control. Biol. Cybern. 104, 31–51. 10.1007/s00422-010-0416-4 21327829

[B37] Georgoulis A. D. Moraiti C. Ristanis S. Stergiou N. (2006). A novel approach to measure variability in the anterior cruciate ligament deficient knee during walking: the use of the approximate entropy in orthopaedics. J. Clin. Monit. Comput. 20, 11–18. 10.1007/s10877-006-1032-7 16523229

[B38] Govindan R. B. Wilson J. D. Eswaran H. Lowery C. L. Preißl H. (2007). Revisiting sample entropy analysis. Phys. A Stat. Mech. its Appl. 376, 158–164. 10.1016/j.physa.2006.10.077

[B39] Gribble P. A. Hertel J. Denegar C. R. Buckley W. E. (2004). The effects of fatigue and chronic ankle instability on dynamic postural control. J. Athl. Train. 39, 321–329. 10.3844/pisp.2010.22.26 15592604 PMC535524

[B40] Hackshaw A. (2008). Small studies: strengths and limitations. Eur. Respir. J. 32, 1141–1143. 10.1183/09031936.00136408 18978131

[B41] Heil J. Büsch D. (2022). Dynamische Haltungskontrolle und körperliche Belastung: ansatz zur Ermittlung des Verletzungsrisikos unter realen sportlichen Bedingungen. Ger. J. Exerc. Sport Res. 53, 196–205. 10.1007/s12662-022-00833-y

[B42] Hespanhol Junior L. C. Pena Costa L. O. Lopes A. D. (2013). Previous injuries and some training characteristics predict running-related injuries in recreational runners: a prospective cohort study. J. Physiother. 59, 263–269. 10.1016/S1836-9553(13)70203-0 24287220

[B43] Hreljac A. (2005). Etiology, prevention, and early intervention of overuse injuries in runners: a biomechanical perspective. Phys. Med. Rehabil. Clin. N. Am. 16, 651–667. 10.1016/j.pmr.2005.02.002 16005398

[B44] Hu K. Ivanov P. C. Chen Z. Carpena P. Stanley H. E. (2001). Effect of trends on detrended fluctuation analysis. Phys. Rev. E - Stat. Phys. Plasmas, Fluids, Relat. Interdiscip. Top. 64, 011114. 10.1103/PhysRevE.64.011114 11461232

[B45] Huygaerts S. Cos F. Cohen D. D. Calleja-González J. Guitart M. Blazevich A. J. (2020). Mechanisms of hamstring strain injury: interactions between fatigue, muscle activation and function. Sports 8, 65–15. 10.3390/sports8050065 32443515 PMC7281534

[B46] Impellizzeri F. M. Rampinini E. Coutts A. J. Sassi A. Marcora S. M. (2004). Use of RPE-based training load in soccer. Med. Sci. Sports Exerc. 36, 1042–1047. 10.1249/01.MSS.0000128199.23901.2F 15179175

[B47] Jenkins P. Earle-Richardson G. Slingerland D. T. May J. (2002). Time dependent memory decay. Am. J. Ind. Med. 41, 98–101. 10.1002/ajim.10035 11813214

[B48] Kaufman C. Berg K. Noble J. Thomas J. (2006). Ratings of perceived exertion of ACSM exercise guidelines in individuals varying in aerobic fitness. Res. Q. Exerc. Sport 77, 122–130. 10.1080/02701367.2006.10599338 16646359

[B49] Kiefer A. W. Ford K. R. Paterno M. V. Schmitt L. C. Myer G. D. Riley M. A. (2013). Inter-segmental postural coordination measures differentiate athletes with ACL reconstruction from uninjured athletes. Gait Posture 37, 149–153. 10.1016/j.gaitpost.2012.05.005 23219784 PMC3556179

[B50] Kodama K. Yasuda K. Akatsuka T. Kuznetsov N. A. Iwata H. (2022). The influence of a vibrotactile biofeedback system on postural dynamics during single-leg standing in healthy older adults. Neurosci. Lett. 786, 136807. 10.1016/j.neulet.2022.136807 35850321

[B51] Ksoll K. S. H. Cotic M. Schmalzl K. Beitzel K. Achtnich A. Imhoff A. (2022). Movement coordination during functional single-leg squat tests in healthy, recreational athletes. Symmetry (Basel) 14, 388. 10.3390/sym14020388

[B52] Kuznetsov N. Bonnette S. Gao J. Riley M. A. (2013). Adaptive fractal analysis reveals limits to fractal scaling in center of pressure trajectories. Ann. Biomed. Eng. 41, 1646–1660. 10.1007/s10439-012-0646-9 22956160

[B107] Latash M. L. (2012). The bliss (not the problem) of motor abundance (not redundancy). Exp. Brain Res. 217, 1–5. 10.1007/s00221-012-3000-4 22246105 PMC3532046

[B53] Liddy J. Busa M. (2023). Considerations for applying entropy methods to temporally correlated stochastic datasets. Entropy 25, 306–321. 10.3390/e25020306 36832672 PMC9955719

[B54] Liddy J. J. Haddad J. M. (2018). Evenly spaced Detrended Fluctuation Analysis: selecting the number of points for the diffusion plot. Phys. A Stat. Mech. its Appl. 491, 233–248. 10.1016/j.physa.2017.08.099

[B55] Liebovitch L. S. Yang W. (1997). Transition from persistent to antipersistent correlation in biological systems. Phys. Rev. E - Stat. Phys. Plasmas, Fluids, Relat. Interdiscip. Top. 56, 4557–4566. 10.1103/PhysRevE.56.4557

[B56] Lin D. Nussbaum M. A. Seol H. Singh N. B. Madigan M. L. Wojcik L. A. (2009). Acute effects of localized muscle fatigue on postural control and patterns of recovery during upright stance: influence of fatigue location and age. Eur. J. Appl. Physiol. 106, 425–434. 10.1007/s00421-009-1026-5 19306019

[B57] Lopes A. D. Hespanhol L. C. Yeung S. S. Costa L. O. P. (2012). What are the main running-related musculoskeletal injuries? Sport. Med. 42, 891–905. 10.1007/bf03262301 PMC4269925 22827721

[B58] Lourenço T. F. Martins L. E. B. Tessuti L. S. Brenzilofer R. Macedo D. V. (2011). Reproducibility of an incremental treadmill Vo2max test with gas exchange analysis for runners. J. Strength Cond. Res. 25, 1994–1999. 10.1519/JSC.0b013e3181e501d6 21487313

[B59] Lun V. Meeuwisse W. H. Stergiou P. Stefanyshyn D. (2004). Relation between running injury and static lower limb alignment in recreational runners. Br. J. Sports Med. 38, 576–580. 10.1136/bjsm.2003.005488 15388542 PMC1724945

[B60] McGregor S. J. Armstrong W. J. Yaggie J. A. Bollt E. M. Parshad R. Bailey J. J. (2011). Lower extremity fatigue increases complexity of postural control during a single-legged stance. J. Neuroeng. Rehabil. 8, 43. 10.1186/1743-0003-8-43 21816084 PMC3239328

[B61] McQuarrie A. D. (1999). A small-sample correction for the Schwarz SIC model selection criterion. Stat. Probab. Lett. 44, 79–86. 10.1016/S0167-7152(98)00294-6

[B62] Messier S. P. Martin D. F. Mihalko S. L. Ip E. DeVita P. Cannon D. W. (2018). A 2-year prospective cohort study of overuse running injuries: the runners and injury longitudinal study (TRAILS). Am. J. Sports Med. 46, 2211–2221. 10.1177/0363546518773755 29791183

[B63] Monjo F. Terrier R. Forestier N. (2015). Muscle fatigue as an investigative tool in motor control: a review with new insights on internal models and posture-movement coordination. Hum. Mov. Sci. 44, 225–233. 10.1016/j.humov.2015.09.006 26406972

[B64] Montull L. Vázquez P. Rocas L. Hristovski R. Balagué N. (2020). Flow as an embodied state. Informed awareness of slackline walking. Front. Psychol. 10, 2993–3011. 10.3389/fpsyg.2019.02993 31998205 PMC6968164

[B65] Mulvad B. Nielsen R. O. Lind M. Ramskov D. (2018). Diagnoses and time to recovery among injured recreational runners in the RUN CLEVER trial. PLoS One 13, 02047422–e204811. 10.1371/journal.pone.0204742 PMC6193581 30312310

[B66] Murnaghan C. D. Squair J. W. Chua R. Inglis J. T. Carpenter M. G. (2014). Cortical contributions to control of posture during unrestricted and restricted stance. J. Neurophysiol. 111, 1920–1926. 10.1152/jn.00853.2012 24523526

[B67] National Safety Council (2022). Sports and recreational injury. Available at: https://injuryfacts.nsc.org/home-and-community/safety-topics/sports-and-recreational-injuries/.

[B68] Paterno M. V. Kiefer A. W. Bonnette S. Riley M. A. Schmitt L. C. Ford K. R. (2015). Prospectively identified deficits in sagittal plane hip-ankle coordination in female athletes who sustain a second anterior cruciate ligament injury after anterior cruciate ligament reconstruction and return to sport. Clin. Biomech. 30, 1094–1101. 10.1016/j.clinbiomech.2015.08.019 PMC4674344 26416200

[B69] Peng C. K. Buldyrev S. V. Havlin S. Simons M. Stanley H. E. Goldberger A. L. (1994). Mosaic organization of DNA nucleotides. Phys. Rev. E 49, 1685–1689. 10.1103/physreve.49.1685 9961383

[B70] Pethick J. Winter S. L. Burnley M. (2015). Fatigue reduces the complexity of knee extensor torque fluctuations during maximal and submaximal intermittent isometric contractions in man. J. Physiol. 593, 2085–2096. 10.1113/jphysiol.2015.284380 25664928 PMC4405761

[B71] Pethick J. Winter S. L. Burnley M. (2016). Loss of knee extensor torque complexity during fatiguing isometric muscle contractions occurs exclusively above the critical torque. Am. J. Physiol. - Regul. Integr. Comp. Physiol. 310, R1144–R1153. 10.1152/ajpregu.00019.2016 27101290

[B72] Petushek E. Nilstad A. Bahr R. Krosshaug T. (2021). Drop jump? single-leg squat? not if you aim to predict anterior cruciate ligament injury from real-time clinical assessment: a prospective cohort study involving 880 elite female athletes journal of orthopaedic and sports physical therapy. J. Orthop. Sports Phys. Ther. 51, 372–378. 10.2519/jospt.2021.10170 34192883

[B73] Pol R. Hristovski R. Medina D. Balague N. (2018). From microscopic to macroscopic sports injuries. Applying the complex dynamic systems approach to sports medicine: a narrative review. Br. J. Sports Med. 53, 1214–1220. 10.1136/bjsports-2016-097395 29674346

[B74] Pollock M. L. Gaesser G. A. Butcher J. D. Després J.-P. Dishman R. K. Franklin B. A. (1998). ACSM Position Stand: the recommended quantity and quality of exercise for developing and maintaining cardiorespiratory and muscular fitness, and flexibility in healthy adults. Med. Sci. Sport. Exerc 30, 975–991. 10.1097/00005768-199806000-00032 9624661

[B75] Quammen D. Cortes N. Van Lunen B. L. Lucci S. Ringleb S. I. Onate J. (2012). Two different fatigue protocols and lower extremity motion patterns during a stop-jump task. J. Athl. Train. 47, 32–41. 10.4085/1062-6050-47.1.32 22488228 PMC3418112

[B76] Quatman-Yates C. C. Bonnette S. Hugentobler J. A. Médé B. Kiefer A. W. Kurowski B. G. (2015). Postconcussion postural sway variability changes in youth: the benefit of structural variability analyses. Pediatr. Phys. Ther. 27, 316–327. 10.1097/PEP.0000000000000193 26397071 PMC4864002

[B77] Quirino J. Santos T. R. T. Okai-Nóbrega L. A. de Araújo P. A. Carvalho R. Ocarino J. de M. (2021). Runners with a history of injury have greater lower limb movement regularity than runners without a history of injury. Sport. Biomech. 00, 1–13. 10.1080/14763141.2021.1929435 34121609

[B78] Ramdani S. Seigle B. Lagarde J. Bouchara F. Bernard P. L. (2009). On the use of sample entropy to analyze human postural sway data. Med. Eng. Phys. 31, 1023–1031. 10.1016/j.medengphy.2009.06.004 19608447

[B79] Rees D. Younis A. MacRae S. (2019). Is there a correlation in frontal plane knee kinematics between running and performing a single leg squat in runners with patellofemoral pain syndrome and asymptomatic runners? Clin. Biomech. 61, 227–232. 10.1016/j.clinbiomech.2018.12.008 30634094

[B80] Riccio G. E. (1993). in Variability and motor control. Editors Newell, K. M. Corcos D. M. (Champaign, IL: Human Kinetics).

[B81] Riccio G. E. Stoffregen T. A. (1988). Affordances as constraints on the control of stance. Hum. Mov. Sci. 7, 265–300. 10.1016/0167-9457(88)90014-0

[B82] Richman J. S. Moorman J. R. (2000). Physiological time-series analysis using approximate entropy and sample entropy. Am. J. Physiol. Hear. Circ. Physiol. 278, H2039–H2049. 10.1152/ajpheart.2000.278.6.H2039 10843903

[B83] Riley M. A. Mitra S. Stoffregen T. A. Turvey M. T. (1997a). Influences of body lean and vision on unperturbed postural sway. Mot. Control 1, 229–246. 10.1123/mcj.1.3.229

[B84] Riley M. A. Wong S. Mitra S. Turvey M. T. (1997b). Common effects of touch and vision on postural parameters. Exp. Brain Res. 117, 165–170. 10.1007/s002210050211 9386016

[B85] Roerdink M. De Haart M. Daffertshofer A. Donker S. F. Geurts A. C. H. Beek P. J. (2006). Dynamical structure of center-of-pressure trajectories in patients recovering from stroke. Exp. Brain Res. 174, 256–269. 10.1007/s00221-006-0441-7 16685508

[B86] Rohrer B. Hogan N. (2003). Avoiding spurious submovement decompositions: a globally optimal algorithm. Biol. Cybern. 89, 190–199. 10.1007/s00422-003-0428-4 14504938

[B87] Rohrer B. Hogan N. (2006). Avoiding spurious submovement decompositions II: a scattershot algorithm. Biol. Cybern. 94, 409–414. 10.1007/s00422-006-0055-y 16570179

[B88] Schütte K. H. Maas E. A. Exadaktylos V. Berckmans D. Venter R. E. Vanwanseele B. (2015). Wireless tri-axial trunk accelerometry detects deviations in dynamic center of mass motion due to running-induced fatigue. PLoS One 10, 01419577–e142012. 10.1371/journal.pone.0141957 PMC4627812 26517261

[B89] Schütte K. H. Seerden S. Venter R. Vanwanseele B. (2018). Influence of outdoor running fatigue and medial tibial stress syndrome on accelerometer-based loading and stability. Gait Posture 59, 222–228. 10.1016/j.gaitpost.2017.10.021 29080511

[B90] Schwarz N. (1999). Self-reports: how the questions shape the answers. Am. Psychol. 54, 93–105. 10.1037//0003-066x.54.2.93

[B91] Springer B. K. Pincivero D. M. (2009). The effects of localized muscle and whole-body fatigue on single-leg balance between healthy men and women. Gait Posture 30, 50–54. 10.1016/j.gaitpost.2009.02.014 19327999

[B92] Terada M. Bowker S. Thomas A. C. Pietrosimone B. Hiller C. E. Rice M. S. (2015). Alterations in stride-to-stride variability during walking in individuals with chronic ankle instability. Hum. Mov. Sci. 40, 154–162. 10.1016/j.humov.2014.12.004 25553561

[B93] Tochigi Y. Segal N. A. Vaseenon T. Brown T. D. (2012). Entropy analysis of tri-axial leg acceleration signal waveforms for measurement of decrease of physiological variability in human gait. J. Orthop. Res. 30, 897–904. 10.1002/jor.22022 22144127 PMC3319858

[B94] Ugalde V. Brockman C. Bailowitz Z. Pollard C. D. (2015). Single leg squat test and its relationship to dynamic KneeValgus and injury risk screening. PM R. 7, 229–235. 10.1016/j.pmrj.2014.08.361 25111946

[B95] van den Hoorn W. Kerr G. K. van Dieën J. H. Hodges P. W. (2018). Center of pressure motion after calf vibration is more random in fallers than non-fallers: prospective study of older individuals. Front. Physiol. 9, 273. 10.3389/fphys.2018.00273 29632494 PMC5879095

[B96] Van Emmerik R. E. A. Ducharme S. W. Amado A. C. Hamill J. (2016). Comparing dynamical systems concepts and techniques for biomechanical analysis. J. Sport Heal. Sci. 5, 3–13. 10.1016/j.jshs.2016.01.013 PMC6191988 30356938

[B97] Van Gent R. N. Siem D. Van Middelkoop M. Van Os A. G. Bierma-Zeinstra S. M. A. Koes B. W. (2007). Incidence and determinants of lower extremity running injuries in long distance runners: a systematic review. Br. J. Sports Med. 41, 469–480. 10.1136/bjsm.2006.033548 17473005 PMC2465455

[B98] Van Wegen E. E. H. Van Emmerik R. E. A. Riccio G. E. (2002). Postural orientation: age-related changes in variability and time-to-boundary. Hum. Mov. Sci. 21, 61–84. 10.1016/S0167-9457(02)00077-5 11983434

[B99] Vázquez P. Hristovski R. Balagué N. (2016). The path to exhaustion: time-variability properties of coordinative variables during continuous exercise. Front. Physiol. 7, 37–38. 10.3389/fphys.2016.00037 26913006 PMC4753307

[B100] Verschueren J. Tassignon B. De Pauw K. Proost M. Teugels A. Van Cutsem J. (2020). Does acute fatigue negatively affect intrinsic risk factors of the lower extremity injury risk profile? A systematic and critical review. Sport. Med. 50, 767–784. 10.1007/s40279-019-01235-1 31782066

[B101] Videbæk S. Bueno A. M. Nielsen R. O. Rasmussen S. (2015). Incidence of running-related injuries per 1000 h of running in different types of runners: a systematic review and meta-analysis. Sport. Med. 45, 1017–1026. 10.1007/s40279-015-0333-8 PMC4473093 25951917

[B102] Vuillerme N. Hintzy F. (2007). Effects of a 200 W-15 min cycling exercise on postural control during quiet standing in healthy young adults. Eur. J. Appl. Physiol. 100, 169–175. 10.1007/s00421-007-0419-6 17318648

[B103] Wagenmakers E. J. Farrell S. (2004). AIC model selection using Akaike weights. Psychon. Bull. Rev. 11, 192–196. 10.3758/BF03206482 15117008

[B104] Willwacher S. Kurz M. Robbin J. Thelen M. Hamill J. Kelly L. (2022). Running-related biomechanical risk factors for overuse injuries in distance runners: a systematic review considering injury specificity and the potentials for future research. Sport. Med. 52, 1863–1877. 10.1007/s40279-022-01666-3 PMC9325808 35247202

[B105] Yamato T. P. Saragiotto B. T. Lopes A. D. (2015). A consensus definition of running-related injury in recreational runners: a modified Delphi approach. J. Orthop. Sports Phys. Ther. 45, 375–380. 10.2519/jospt.2015.5741 25808527

[B106] Zech A. Steib S. Hentschke C. Eckhardt H. Pfeifer K. (2012). Effects of localized and general fatigue on static and dynamic postural control in male team handball athletes. J. Strength Cond. Res. 24, 1162–1168. 10.1519/JSC.0b013e31822dfbbb 22446681

