# Supplementary material for: Changes to balance dynamics following a high-intensity run are associated with future injury occurrence in recreational runners
Source: Front Netw Physiol. 2023 Nov 21;3:1227861. doi: 10.3389/fnetp.2023.1227861 (PMC10699445; doi:10.3389/fnetp.2023.1227861)
Supplement: Supplementary file 1 [file Table1.pdf]

frontiers

Supplementary Material

Changes to balance dynamics following a high intensity run are associated with future injury occurrence in recreational runners

Mariana R. C. Aquino[1,2*], Joshua J. Liddy[2], C. Dane Napoli[2], Sergio T. Fonseca[1], Richard E. A. van Emmerik[2], Michael A. Busa[2,3]

[1] Graduate Program of Rehabilitation Sciences, Department of Physical Therapy, Universidade Federal de Minas Gerais, Belo Horizonte, Brazil, 31270-901
[2] Department of Kinesiology, University of Massachusetts Amherst, Amherst, MA, 01003
[3] Center for Human Health and Performance, Institute for Applied Life Sciences, University of Massachusetts Amherst, Amherst, MA, 01003

*** Correspondence:** Mariana R. C. Aquino: mariaquino@ufmg.br

Supplementary Table S1. Training characteristics. Means and standard deviations for training volume and pace at the initial and six-month timepoints for the injured and non-injured groups. The results from the two-way mixed-model ANOVAs are reported, including F-values, p-values, and η^2_p. Post hoc comparisons are reported where applicable. For training volume, there was a significant Time x Group interaction. Bonferroni adjusted post-hoc comparisons showed no statistical differences between groups at either time point (initial: $F_{1,25} = 0.003$; $p = .956$; $\eta^2_p = .05$; 6 months: $F_{1,25} = 2.65$; $p = .12$; $\eta^2_p = .19$) or within groups across time (injured: $F_{1,25} = 2.20$; $p = .151$; $\eta^2_p = .08$; non-injured: $F_{1,25} = 2.68$; $p = .115$; $\eta^2_p = .10$). For training pace, there were no significant effects (all $p >> 0.05$).

	Time	Injured	Non-injured	Statistical effect								
				Time			Group			Time × Group		
		Mean (SD)	Mean (SD)	$F_{1,25}$	p	η^2_p	$F_{1,25}$	p	η^2_p	$F_{1,25}$	p	η^2_p
Volume (km/week)	Initial	42.0 (22.3)	41.4 (26.4)	0.012	.92	.00	0.86	.36	.04	4.87	.037*	.17
	6 months	33.0 (15.1)	51.3 (38.0)									
Pace (min/km)	Initial	5:19 (0:36)	5:29 (0:49)	0.004	.95	.00	0.59	.45	0.2	0.015	.90	.23
	6 months	5:18 (0:33)	5:30 (0:42)									

SD – standard deviation; * - statistical significance ($p < 0.05$)
