# Supplementary material for: Changes to balance dynamics following a high-intensity run are associated with future injury occurrence in recreational runners
Source: Front Netw Physiol. 2023 Nov 21;3:1227861. doi: 10.3389/fnetp.2023.1227861 (PMC10699445; doi:10.3389/fnetp.2023.1227861)
Supplement: Supplementary file 2 [file Presentation1.pdf]

Supplementary Material

Changes to balance dynamics following a high intensity run are associated with future injury occurrence in recreational runners

Mariana R. C. Aquino[1,2*], Joshua J. Liddy[2], C. Dane Napoli[2], Sergio T. Fonseca[1], Richard E. A. van Emmerik[2], Michael A. Busa[2,3]

[1] Graduate Program of Rehabilitation Sciences, Department of Physical Therapy, Universidade Federal de Minas Gerais, Belo Horizonte, Brazil, 31270-901
[2] Department of Kinesiology, University of Massachusetts Amherst, Amherst, MA, 01003
[3] Center for Human Health and Performance, Institute for Applied Life Sciences, University of Massachusetts Amherst, Amherst, MA, 01003

*** Correspondence:** Mariana R. C. Aquino: mariaquino@ufmg.br

Supplementary Video S1. Example of the single-leg squat task performance. This video from the pilot study shows a participant performing a sequence of single-leg squats with the dominant foot aligned perpendicular to a vertical screen, the trunk held in an upright position, and hands behind their head. The laser, which was attached to the thigh cluster, is pointed towards the vertical screen, which contains two yellow, horizontal targets. The goal of the task is to continuously move the laser point between the bottom and top targets with the requirement that the laser enter or pass each target before reversing direction. Participants performed the task uninterrupted for 60 s at a self-selected pace. The test was stopped and repeated if the participant moved their stance foot, touched the ground or stance limb with the contralateral leg or was consistently undershooting the targets. Task performance was assessed by examining vertical body displacements, which was strongly correlated with the laser point displacements, while balance control was assessed with transverse plane center of mass displacements.
