# Supplementary figures and images for: Changes to balance dynamics following a high-intensity run are associated with future injury occurrence in recreational runners
Source: Front Netw Physiol. 2023 Nov 21;3:1227861. doi: 10.3389/fnetp.2023.1227861 (PMC10699445; doi:10.3389/fnetp.2023.1227861)

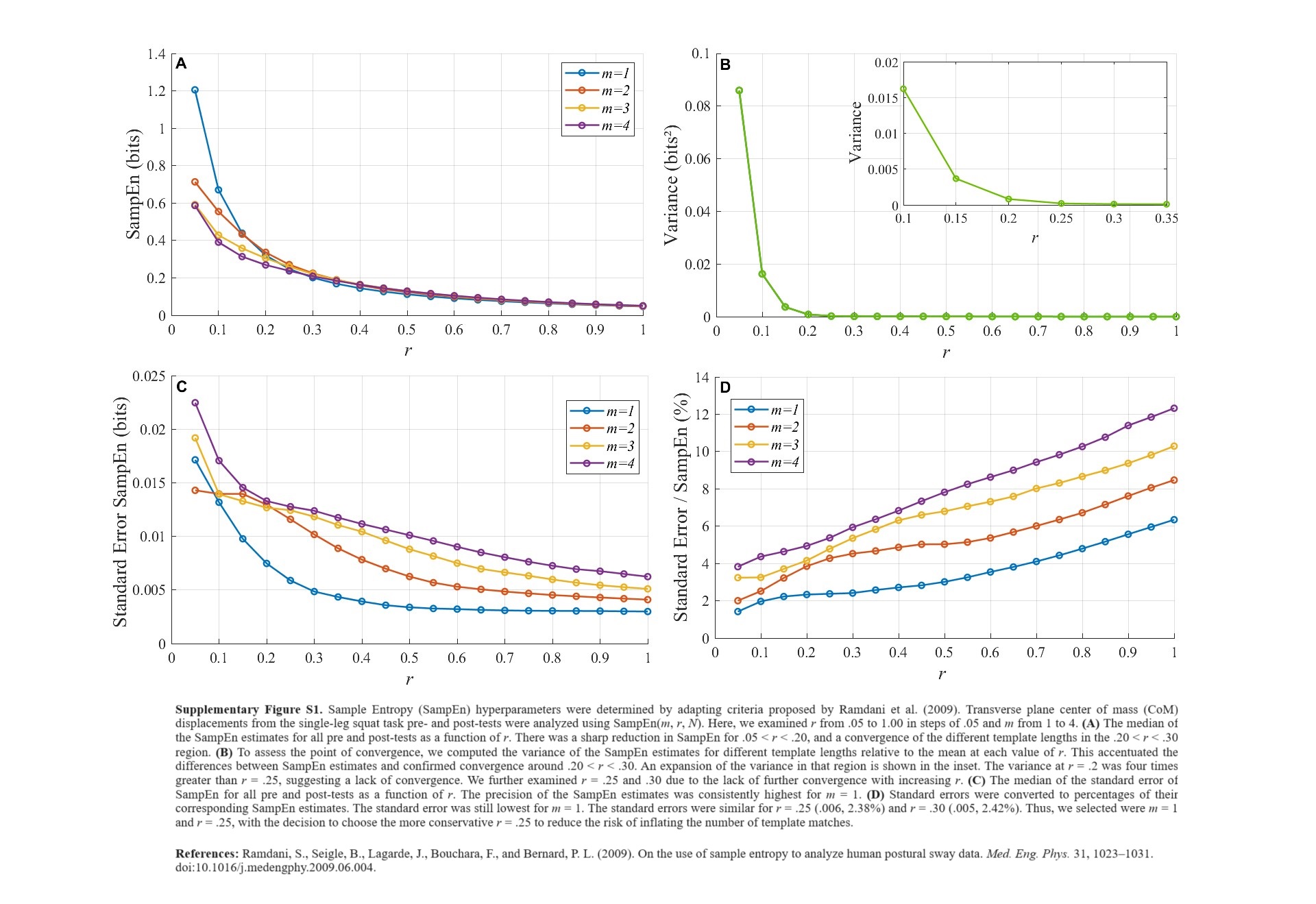

Supplement: Supplementary file 4 [file Image1.jpg]
